# Supplementary material for: Optimized Photoemission from Organic Molecules in 2D Layered Halide Perovskites
Source: J Am Chem Soc. 2026 Jan 13;148(3):3760–74. doi: 10.1021/jacs.5c20638 (PMC12856906; doi:10.1021/jacs.5c20638)
Supplement: Supplementary file 1 [file ja5c20638_si_001.pdf]

Supplementary Information

Optimized Photoemission from Organic Molecules in 2D Layered Halide Perovskites

Muhammad S. Muhammad,[1] Dilruba A. Popy,[1] Hamza Shoukat,[1] John M. Lane,[2] Neeraj Rai,[2] Vojtěch Vaněček,[4] Zdeněk Remeš,[4] Romana Kučerková,[4] Vladimir Babin,[4] Chenjia Mi,[1] Yitong Dong,[1] Mark D. Smith,[3] Novruz G. Akhmedov,[1] Daniel T. Glatzhofer,[1] Bayram Saparov[1*]

[1]*Department of Chemistry & Biochemistry, The University of Oklahoma, Norman, Oklahoma, 73019, United States*
[2]*Dave C. Swalm School of Chemical Engineering and Center for Advanced Vehicular Systems, Mississippi State University, Starkville, Mississippi, 39762, United States*
[3]*Department of Chemistry and Biochemistry, University of South Carolina, Columbia, South Carolina, 29208, United States*
[4]*Institute of Physics, Academy of Science of the Czech Republic, Cukrovarnicka 10, Praha, 16200, Czech Republic*
*Author to whom correspondence should be addressed: saparov@ou.edu

Synthesis of organic salts:

1. **(4-Methylbenzyl)triphenylphosphonium chloride (4):**

A 500 mL round bottom flask was charged with 14.88 g (104.4 mmol) 1-(chloromethyl)-4-methylbenzene, 27.55 g (104.4 mmol) triphenylphosphine, and 150 mL toluene. The flask was fitted with a condenser and the mixture was stirred (magnetic) to dissolve the triphenylphosphine. The resulting clear solution was gently heated to reflux solvent for 6 h. The mixture was allowed to cool to room temperature with continuous stirring overnight and the white solid that formed was collected and dried using suction filtration. The filtrate was returned to the reaction flask, heated to reflux solvent overnight, cooled, and the additional product was again collected and dried using suction filtration. The products were combined to give 20.32 g (48%) of the phosphonium salt, which was used without further purification. **^{1}H-NMR** (400 MHz, $CDCl_3$): 2. 5 (d, *J* = 2.5 Hz, 3H) 5.41 (d, *J* = 14.2 Hz, 2H), 6.92 (m, 2H), 6.95 (m, 2H), 7.63 (m, ^{ortho}H/Ph, 6H), 7.75 (m, $^{meta/para}$H/Ph, 9H)[1]. The coupling constants between ^{31}P and the protons of the CH_2-C_6H_4-CH_3 fragment were confirmed by the iterative procedure, whereas $^nJ_{H,P}$ (n = 3, 4 and 5) is not determined due to the highly overlapping protons of the $(Ph)_3P$ moiety.

The ^{1}H NMR spectrum of compound **4** in $CDCl_3$

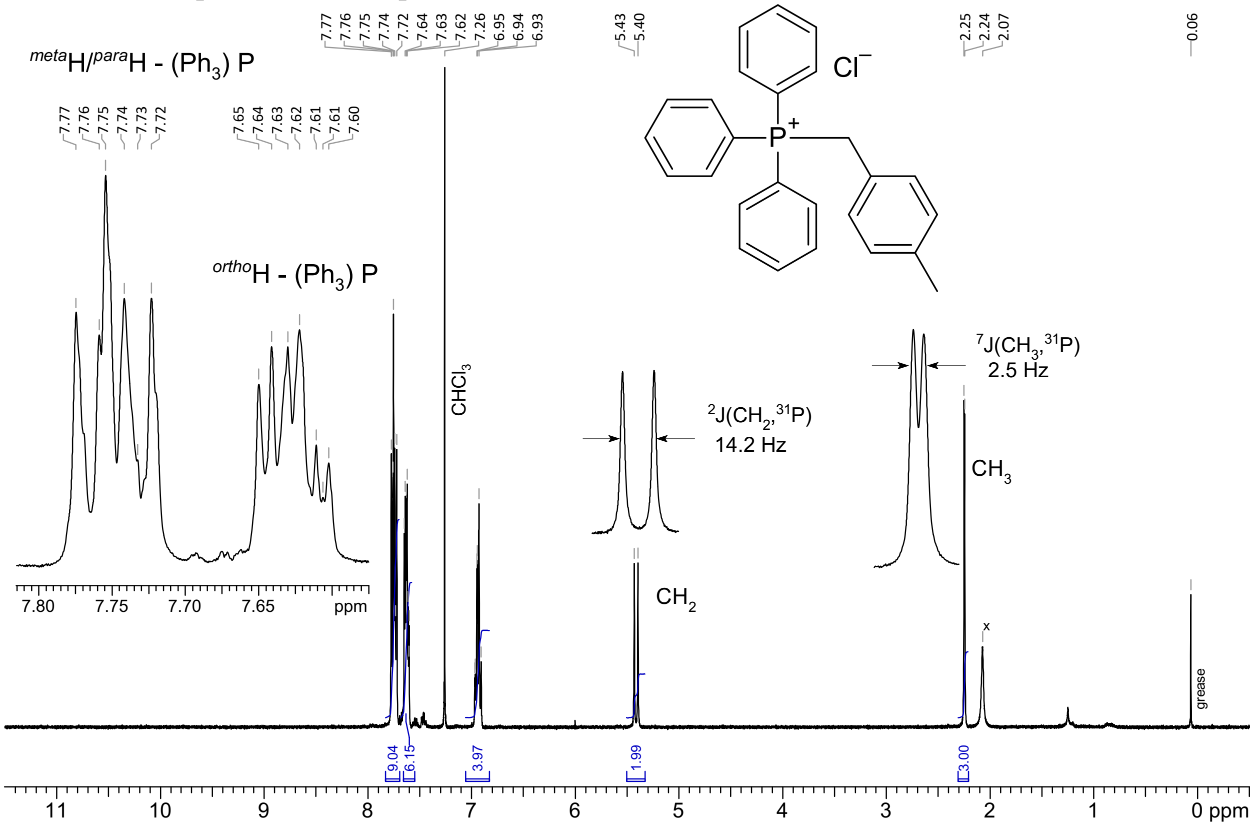

Calculated multiplicity patterns of protons of the CH_2-C6H_4-CH3 fragment of compound **4**. The coupling constants between CH_3 and AA’ and between CH_3 and BB’, as well as between CH_2 and AA’ and between CH_2 and BB’ were not considered in the simulation.

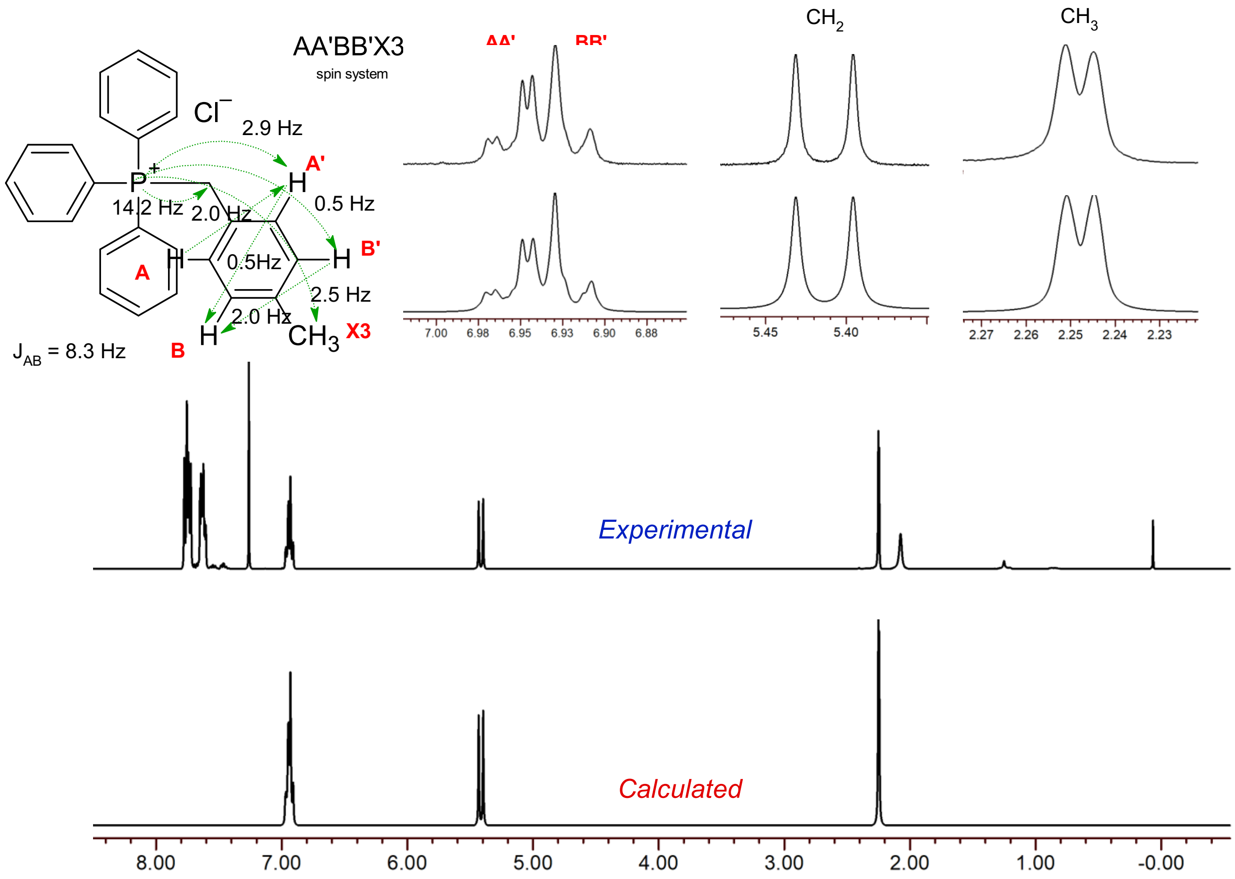

2. **(*E/Z*)-1-bromo-4-(4-methylstyryl)benzene (3a):**

(4-Methylbenzyl)triphenylphosphonium chloride (4) (12.10 g, 30.03 mmol) was added to a 250 mL Erlenmeyer flask. Absolute ethanol (50 mL) and 4-bromobenzaldehyde (5.56 g, 30.0 mmol) were added to the flask, the mixture was stirred (magnetic) with gentle heating on a hot plate until all the solids had dissolved. In a separate flask, 3.37 g (60.0 mmol) potassium hydroxide was dissolved in 50 mL absolute ethanol. The potassium hydroxide solution was added to the aldehyde/phosphonium salt solution in one portion. A clear yellow solution formed initially, followed shortly by precipitation of a white solid. Additional ethanol (25 mL) was added with stirring for 15 mins. The mixture was heated to gently boil for 10-15 m. The mixture was allowed to cool and 100 ml 60% (v/v) ethanol/water was added with stirring. The product was collected by gravity filtration, washed with 60% ethanol/water, and dried using suction to give 5.50 g (67%) of white product **3**. ^{1}H-NMR spectroscopy showed the product to be a ca. 30/70 mixture of the *Z*- and *E*-isomers of stilbene **3**. Separation or isomerization of the mixture was unnecessary as the next reaction produced all *E*-product. **^{1}H-NMR** (400 MHz, $CDCl_3$) *Z*-Isomer: 2.32 (s, 3H), 6.46 (d, J = 12.2 Hz, 1H), 6.60 (d, J = 12.2 Hz, 1H), 7.04 (d, J = 8.2 Hz, 2H), 7.12 (d, J = 8.2 Hz, 2H), 7.12 (d, J = 8.5 Hz, 2H), 7.34 (d, J = 8.5 Hz, 2H).[2] *E*-Isomer: 2.37 (s, 3H), 6.99 (d, J = 16.3 Hz, 1H), 7.07 (d, J = 16.3 Hz, 1H), 7.18 (d, J = 8.2 Hz, 2H), 7.36 (d, J = 8.5 Hz, 2H), 7.41 (d, J = 8.2 Hz, 2H), 7.47 (d, J = 8.5 Hz, 2H).[2]

The ^{1}H NMR spectrum of compound **3a** in $CDCl_3$

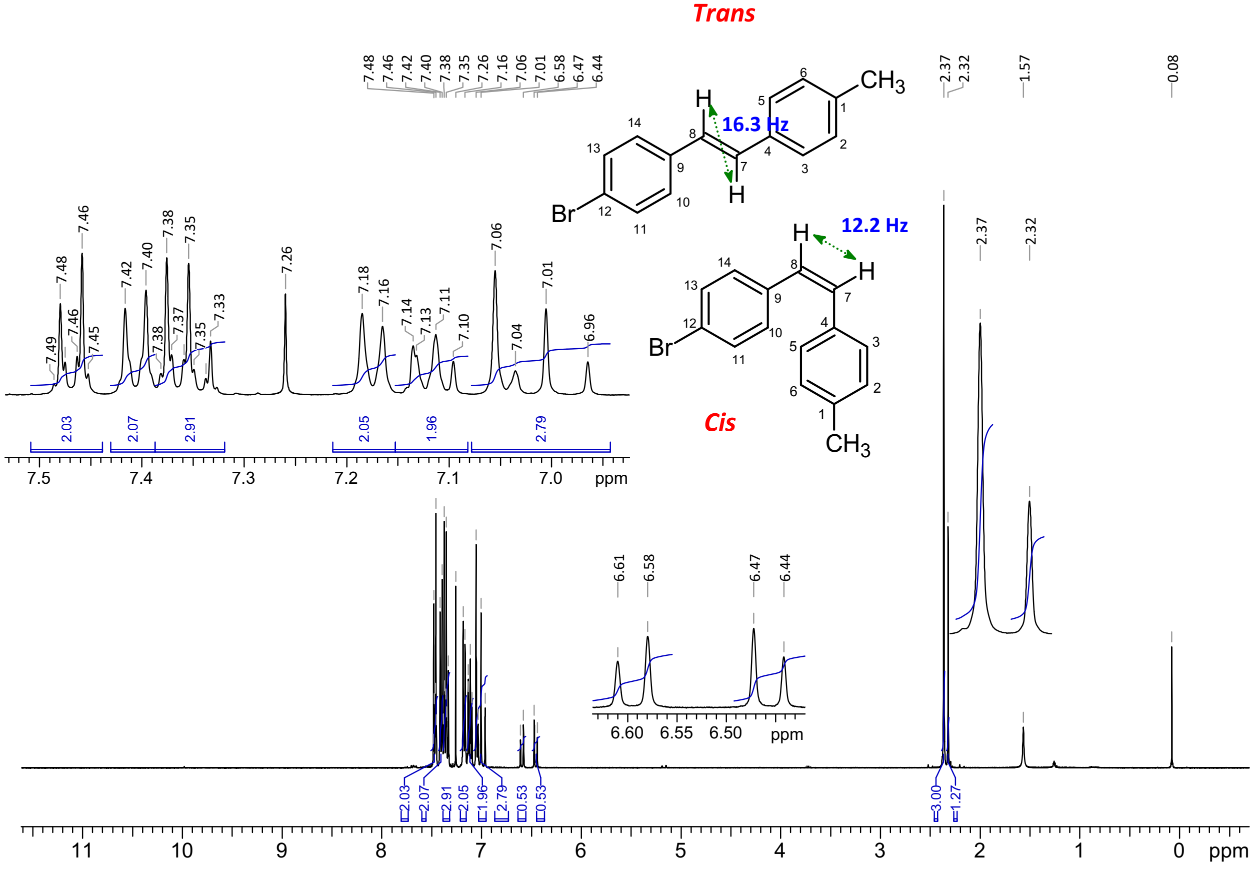

Insets of the NMR parameters (chemical shift δ (ppm) and coupling constants (J/Hz) for all peaks in compound **3a.**

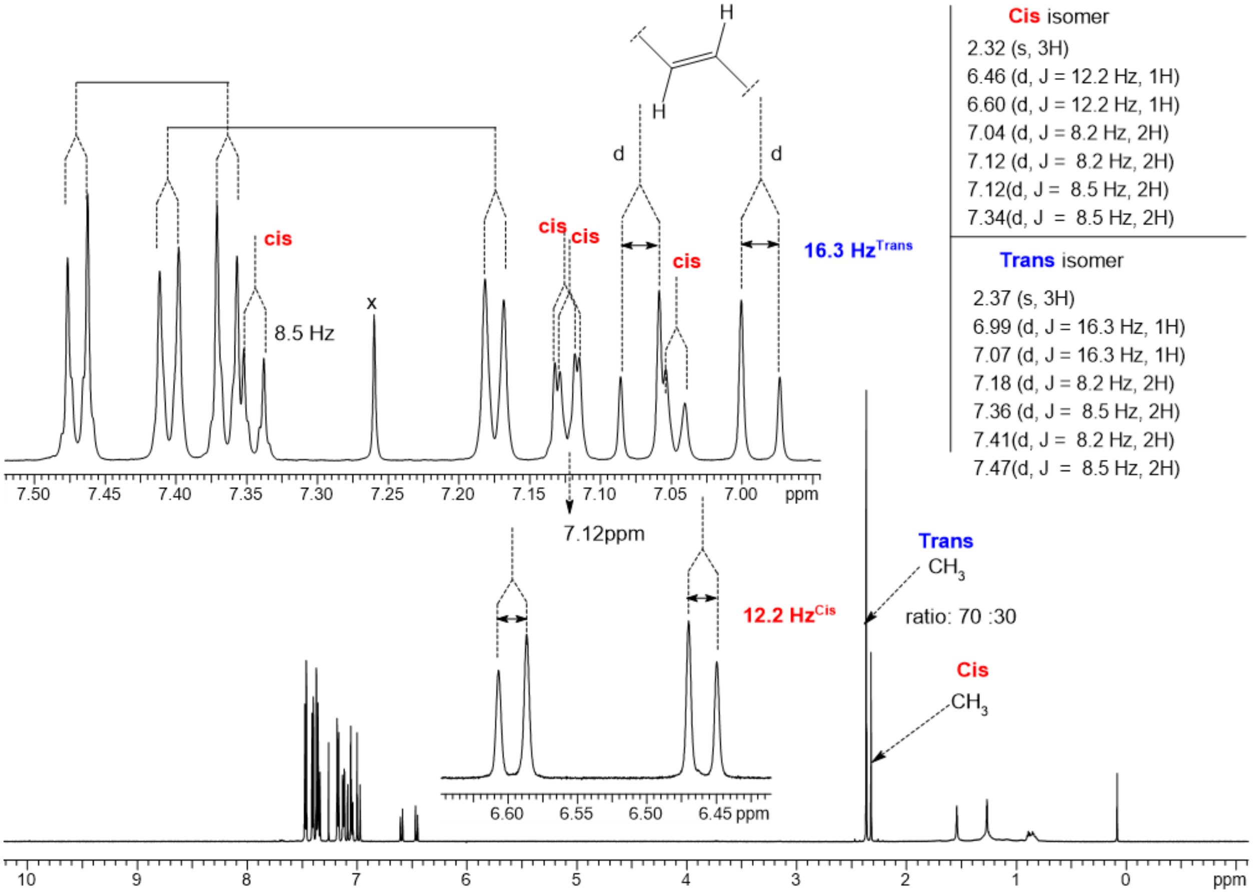

3. **(*E*)-1-bromo-4-(4-(bromomethyl)styryl)benzene (2a):**

A 100 mL round bottom flask was charged with 2.73 g (10.0 mmol) of (*E/Z*)-1-bromo-4-(4-methylstyryl)benzene (3a), 1.90 g (10.0 mmol) N-bromosuccinimide, and 50 mL benzene and fitted with a reflux condenser. Upon warming the mixture, a clear solution was obtained. A catalytic amount of benzoyl peroxide (0.05 g, 200 µmol) was added, and the mixture was heated overnight at 80 °C with stirring (magnetic). The reaction was allowed to cool to room temperature while stirring and a white precipitate typical of succinimide was visible upon cooling. The crude mixture was filtered to remove insoluble succinimide. The filtrate was washed with 5% aqueous sodium carbonate solution four times and dried over magnesium sulfate. The organic solution was carefully decanted and dried under a stream of nitrogen to give 3.00 g (85%) of creamy white solid **2a**. **^{1}H-NMR** (400 MHz, $CDCl_3$) 4.5 (s, 2H), 7.04 (d, J = 16.4 Hz, 1H), 7.07 (d, J = 16.2 Hz, 1H), 7.38 (m, 4H), 7.39 (d, J = 4.0 Hz, 2H), 7.48 (m, 4H).[3]

^{1}H NMR spectrum of compound **2a** in $CDCl_3$

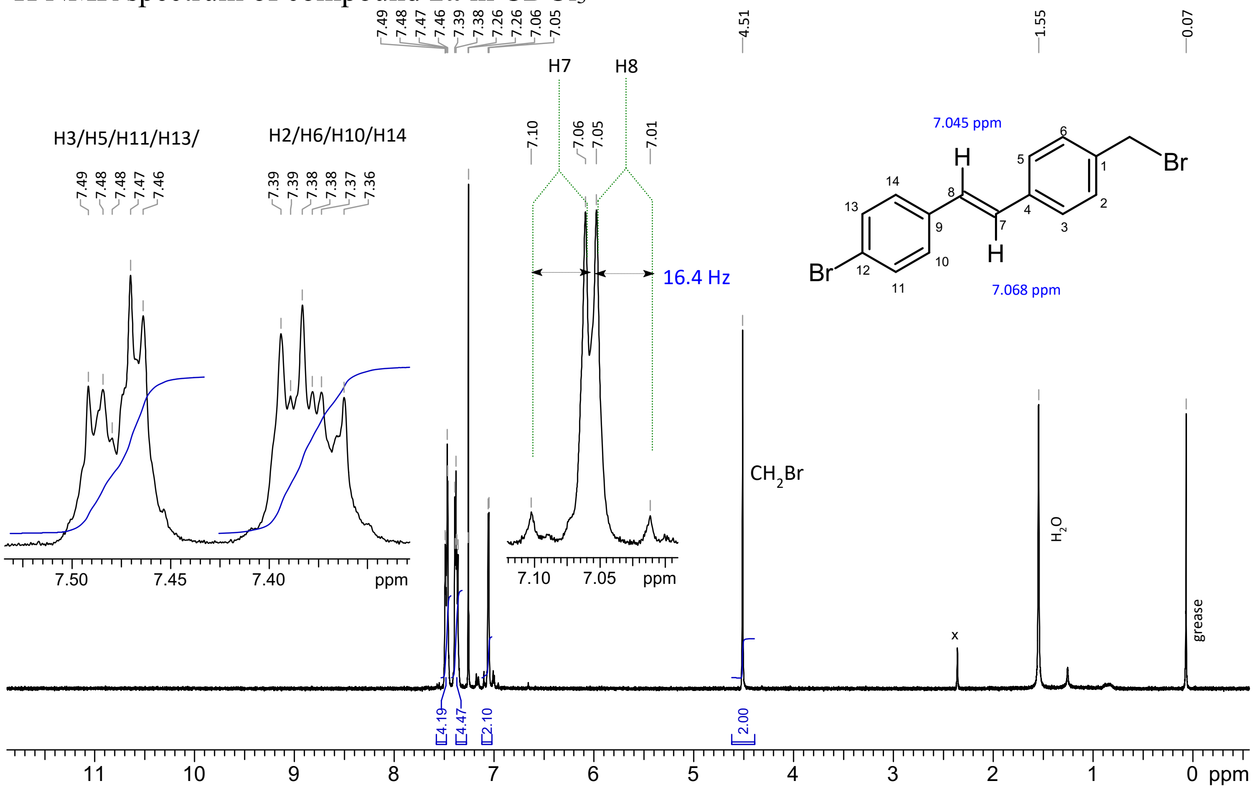

4. **(*E*)-(4-(4-bromostyryl)phenyl)methanaminium chloride (1a):**

(*E*)-1-bromo-4-(4-(bromomethyl)styryl)benzene (**2a**) (1.00 g, 2.84 mmol) was introduced into a 250 mL round bottom flask along with 12 mL of tetrahydrofuran and 9 mL of absolute ethanol. The mixture was warmed to dissolve the stilbene 2a and a solution of 185 mg (2.84 mmol) of sodium azide (NaN_3) in 4 mL distilled water was added to the reaction mixture. The flask was fitted with a condenser and the mixture was heated to gently reflux solvent for 1 h. Triphenylphosphine (0.745 g, 2.84 mmol) was added portion-wise, and the evolution of gas was observed. The mixture was heated continuously until gas evolution stopped. Concentrated aqueous HCl (6 mL) was added and the mixture was heated to reflux solvent for two hours and allowed to cool to room temperature overnight. The product 1a that precipitated was isolated as a yellow solid 1a (0.60 g, 73%). **^{1}H-NMR** (400 MHz, DMSO-d_6) 4.00 (q, *J* = 5.9 Hz, 2H), 7.29 (d, *J* = 16.4, 1H), 7.31 (d, *J* = 16.4 Hz, 1H), 7.51 (d, *J* = 8.3 Hz, 2H), 7.57 (s, 4H), 7.63 (d, *J* = 8.3 Hz, 2H); 8.56 (br s, 3H

The ^{1}H NMR spectrum of compound **1a** in DMSO.

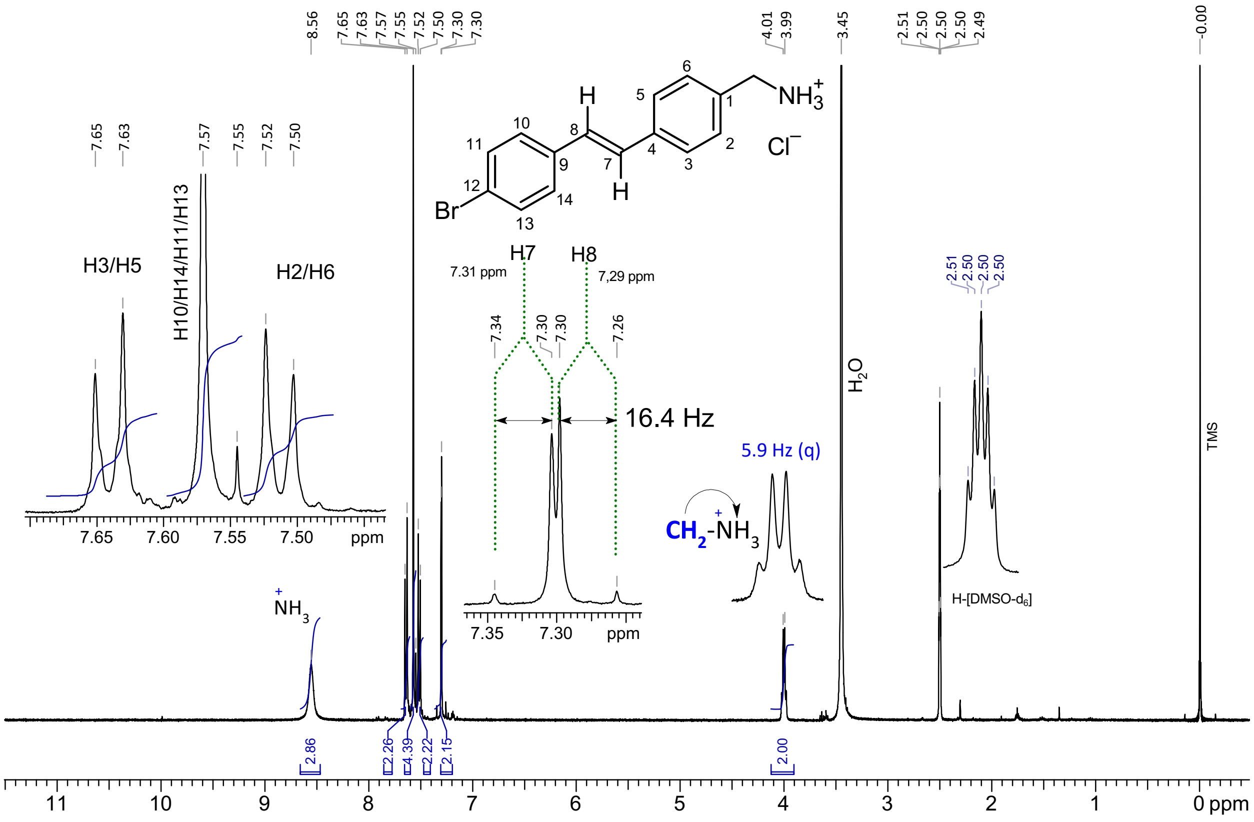

5. **(*E*)-1-methyl-4-styrylbenzene(3b):**

(4-Methylbenzyl)triphenylphosphonium chloride 6.00 g (14.9 mmol) was added to a 250 mL Erlenmeyer flask. Absolute ethanol (25 mL) and benzaldehyde (1.58 g, 14.9 mmol) were added to the flask, the mixture was stirred (magnetic) with gentle heating on a hot plate until all the solids had dissolved. In a separate flask, 1.19 g (29.8 mmol) of sodium hydroxide was dissolved in 25 mL of absolute ethanol. The sodium hydroxide solution was added to the aldehyde/phosphonium salt solution in one portion. A clear yellow solution formed initially, followed shortly by precipitation of a white solid. Additional ethanol (25 mL) was added with stirring for 15 mins. The mixture was heated to gently boil for 10-15 m. The mixture was allowed to cool, and 100 ml 60% (v/v) ethanol/water was added with stirring. The product was collected by gravity filtration, washed with 60% ethanol/water, and dried using suction to give 0.85 g (30%) of white product 3b **^{1}H-NMR** (400 MHz, $CDCl_3$) 2.37 (s, 3H), 7.06 (d, J = 16.4 Hz, 1H), 7.10 (d, J = 16.4 Hz, 1H), 7.17 (d, J = 7.6 Hz, 2H), 7.25 (m, 1H), 7.35 (t, J = 7.2 Hz, 2H), 7.42, (d, J = 8.4 Hz, 2H), 7.51 (d, J = 8.4 Hz, 2H).[2] The ^{1}H NMR spectrum reveals high-order splitting patterns for the phenyl and CH_3-C_6H_4 moieties of compound **3b**. An iterative procedure was used to extract all spin–spin coupling constants.

The ^{1}H NMR spectrum of compound **3b** in $CDCl_3$

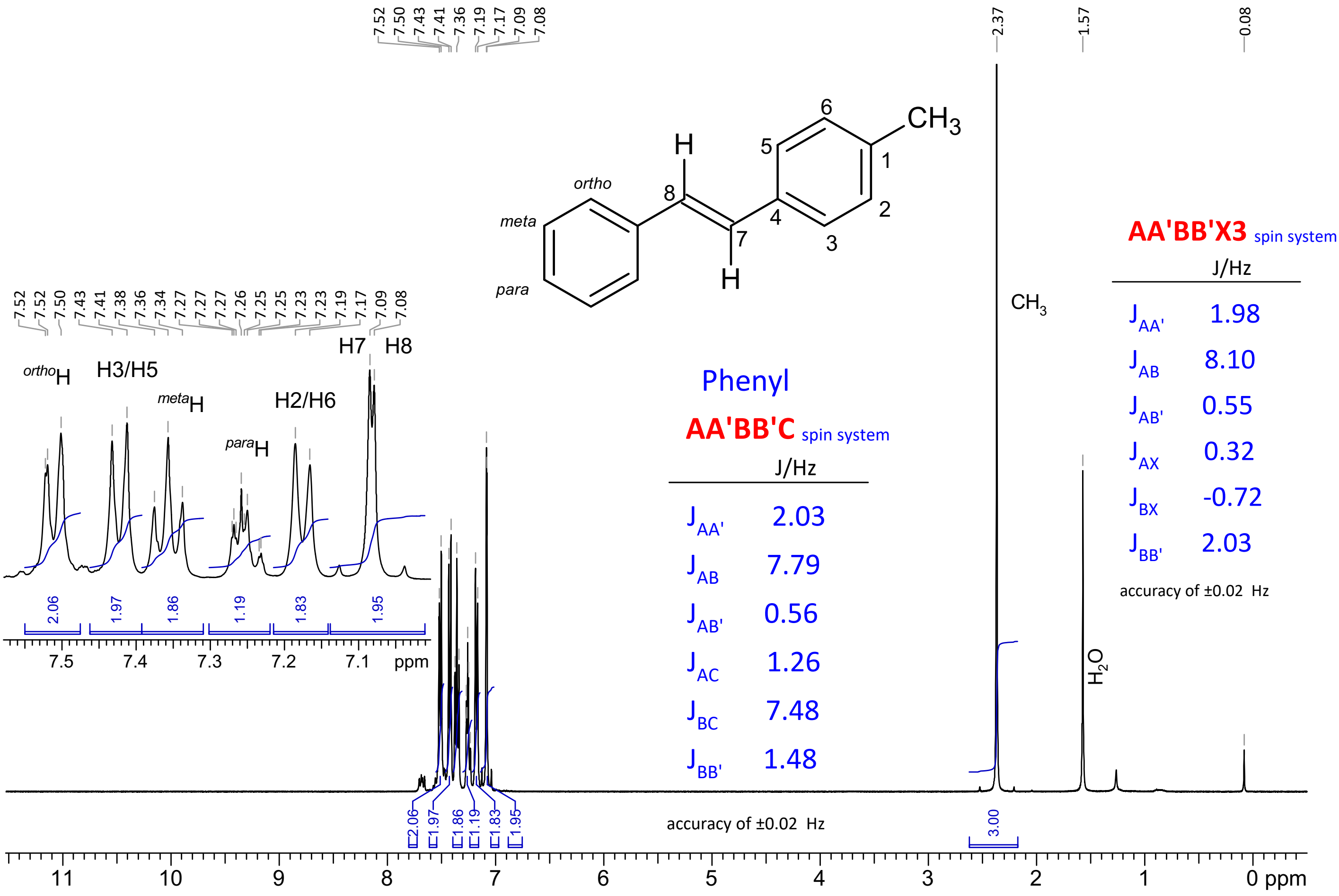

Calculated and experimental second order splitting patterns observed in the NMR spectrum of compound **3b**.

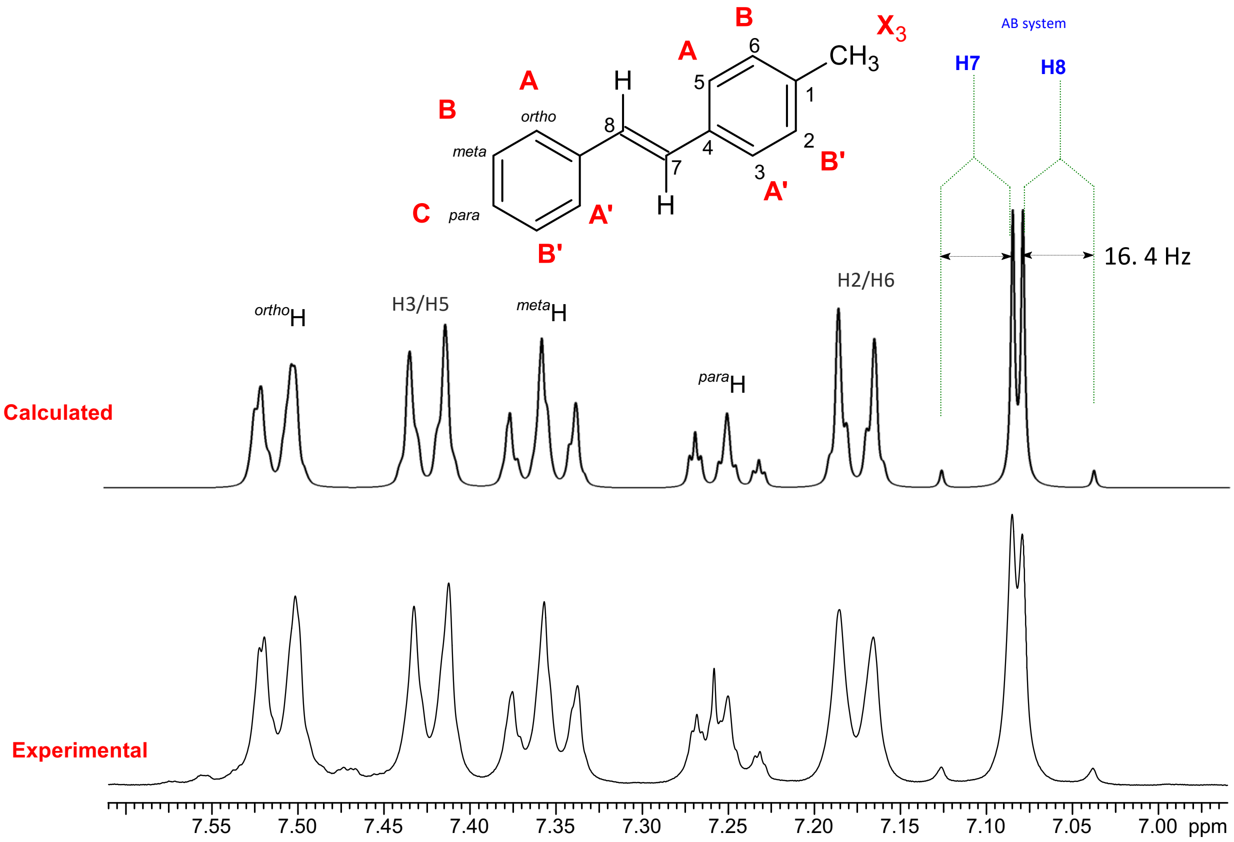

6. **(*E*)-1-(bromomethyl)-4-styrylbenzene(2b):**

A 250 mL round bottom flask was charged with 1.50 g (7.75 mmol) of (*E*)-1-methyl-4-styrylbenzene (3b), 1.51 g (8.49 mmol) of N-bromosuccinimide and 38 mL of benzene and fitted with a reflux condenser. Upon warming the mixture, a clear solution was obtained. A catalytic amount of benzoyl peroxide (0.037 mg 155 μmol) was added, and the mixture was heated overnight at 80 °C with stirring (magnetic). The reaction was allowed to cool to room temperature while stirring and a white precipitate typical of succinimide was visible upon cooling. The crude mixture was filtered to remove insoluble succinimide. The filtrate was washed with 5% aqueous sodium carbonate solution four times and dried over magnesium sulfate. The organic solution was carefully decanted and dried under a stream of nitrogen to give 2.017 g (80 %) of yellow solid **2b**. ^{1}**H-NMR** (400 MHz, $CDCl_3$) 4.52 (s, 2H), 7.10 (d, *J* = 16.4 Hz, 1H), 7.12 (d, *J* = 16.4 Hz, 1H), 7.27 (m, ^{para}H/Ph), 7.37 (m, ^{meta}H/Ph +H2/H6, 4H), 7.51 (m, ^{ortho}H/Ph +H3/H5, 4H)[4,5]

The ^{1}H NMR spectrum of compound **2b** in $CDCl_3$

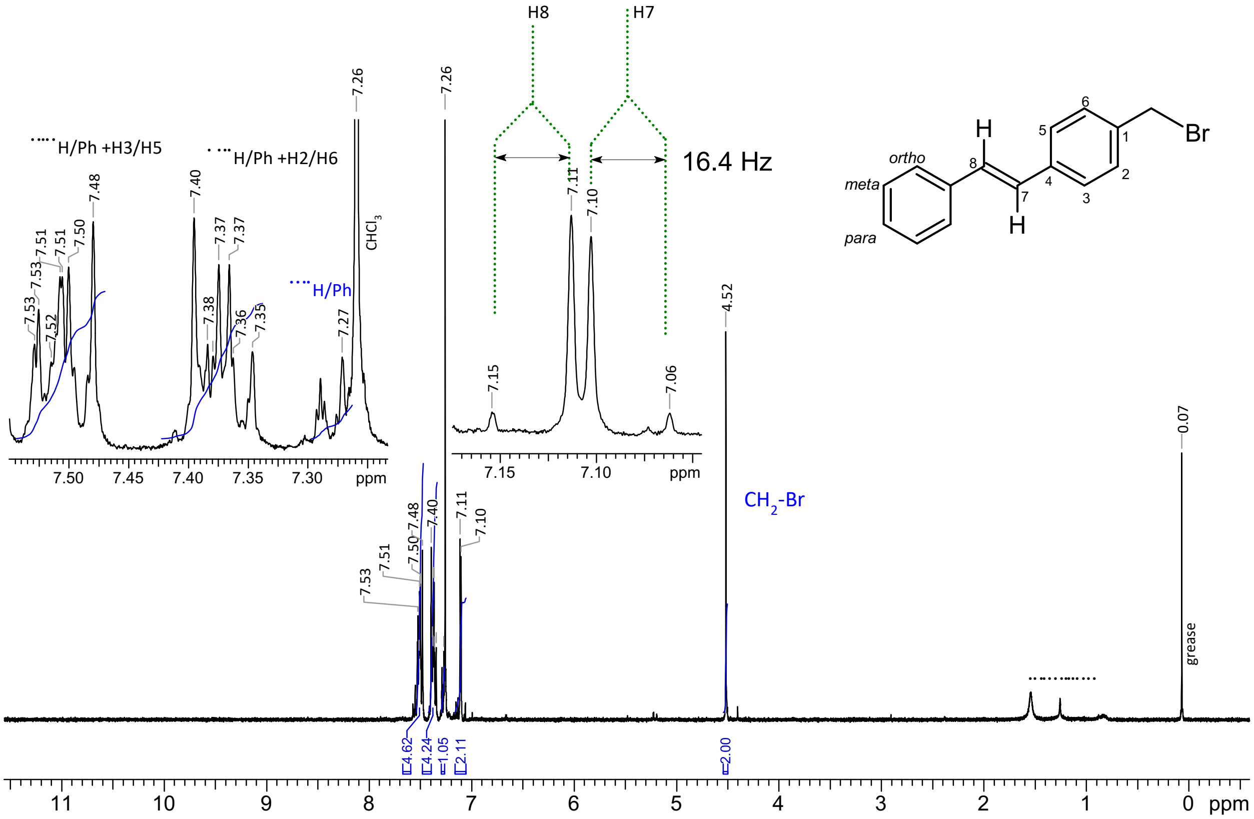

7. **(*E*)-(4-styrylphenyl)methanaminium chloride (1b):**

(*E*)-1-(bromomethyl)-4-styrylbenzene (2b) (1.50 g, 5.49 mmol) was introduced into a 250 mL round bottom flask along with 18 mL of tetrahydrofuran and 13 mL of absolute ethanol. The mixture was warmed to dissolve the stilbene **2b**, and a solution of 357 mg (5.49 mmol) of sodium azide (NaN_3) in 6 mL distilled water was added to the reaction mixture. The flask was fitted with a condenser and the mixture was heated to gently reflux solvent for 1 h. Triphenylphosphine (1.44 g, 5.49 mmol) was added portion-wise, and the evolution of gas was observed. The mixture was heated continuously until gas evolution stopped. Concentrated aqueous HCl (9 mL) was added, and the mixture was heated to reflux solvent for two hours and allowed to cool to room temperature overnight. The product 1b that precipitated was isolated as a yellow solid 1b (0.60 g 73 %). **^{1}H-NMR** (400 MHz, DMSO-d_6) 4.0 (q, J = 5.6 Hz, 2H), 7.24 (d, J = 16.4 Hz, 1H), 7.29 (d, J = 16.4 Hz, 1H), 7.28 (t, 1H), 7.37 (t, J = 7.2, 2H), 7.47 (d, J = 8.0 Hz, 2H), 7.59 (d, J = 8.0 Hz, 2H), 7.63 (d, J = 8.0 Hz, 2H).[6] An iterative procedure was used to extract all spin–spin coupling constants for the phenyl and CH_3-C_6H_4 moieties.

The ^{1}H NMR spectrum of compound **1b** in $CDCl_3$

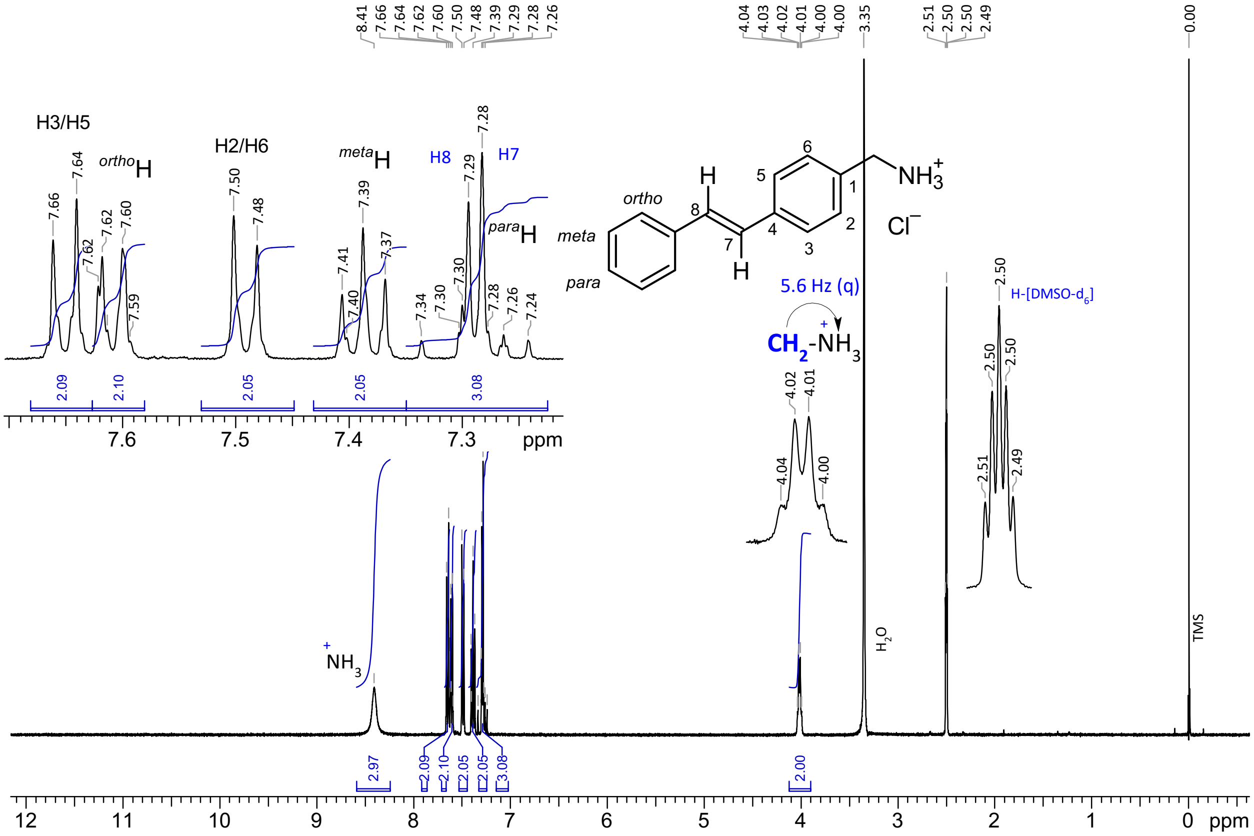

Calculated and experimental second-order splitting patterns observed in the NMR spectrum of compound **1b**.

Phenyl
AA'BB'C spin system

	J/Hz
$J_{AA'}$	1.98
J_{AB}	7.81
$J_{AB'}$	0.54
J_{AC}	1.27
J_{BC}	7.48
$J_{BB'}$	1.49

AA'BB'X2 spin system

$J_{AA'}$	1.97
J_{AB}	8.20
$J_{AB'}$	0.58
J_{AC}	1.27
J_{BC}	7.49
$J_{BB'}$	1.48
J_{AX}	0.32
J_{BX}	-0.72

accuracy of ±0.02 Hz

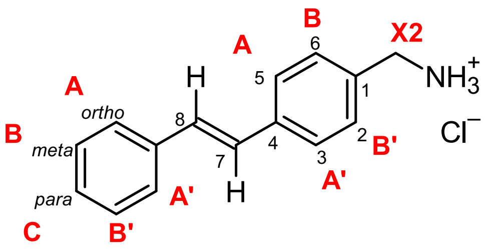

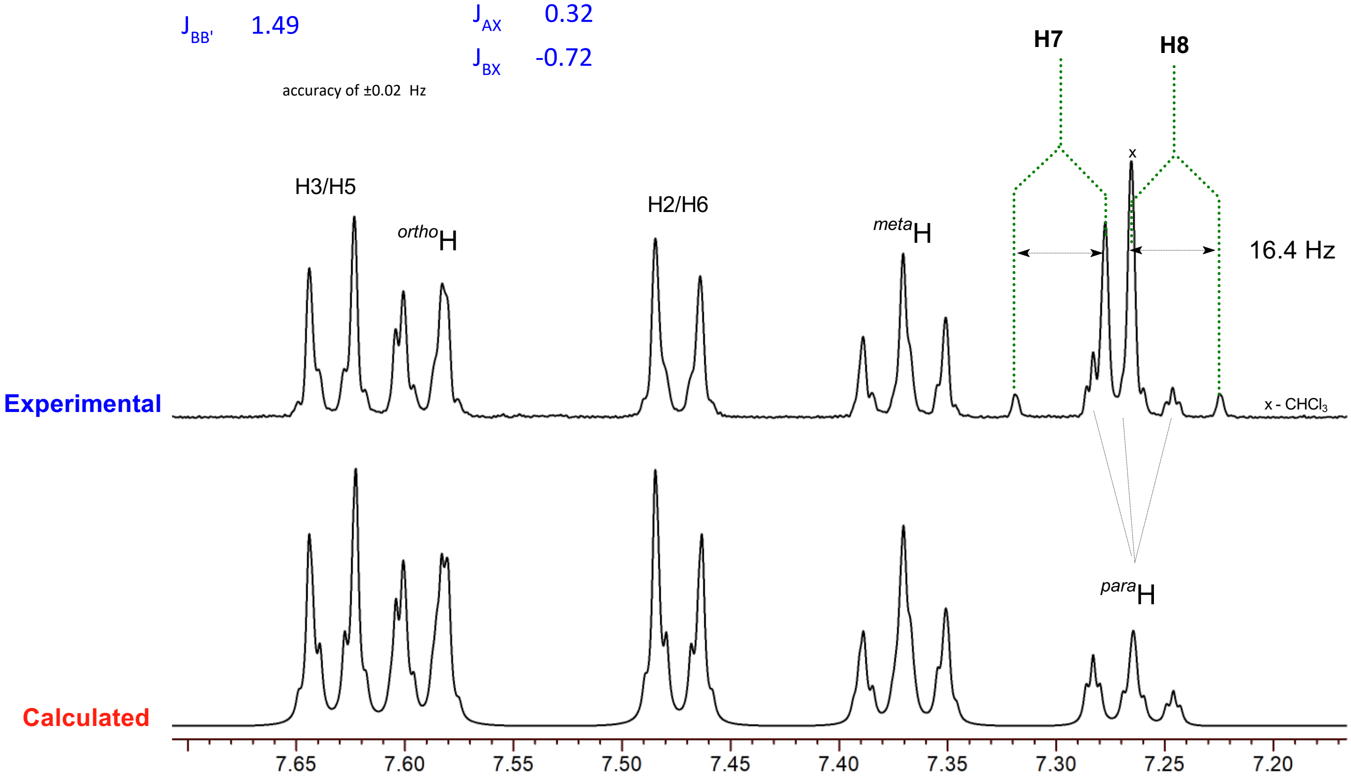

Single crystal X-ray crystallography

$(C_{15}H_{16}N)_2CdCl_4$: The compound crystallizes in the orthorhombic system. The pattern of systematic absences in the intensity data was uniquely consistent with the space group *Pbca*, which was confirmed by structure solution. The asymmetric unit consists of one $C_{15}H_{16}N^+$ cation, two chloride anion and (formally) half of one cadmium atom, which is located in a crystallographic inversion center. All non-hydrogen atoms were refined with anisotropic displacement parameters. Hydrogen atoms bonded to carbon were located in difference Fourier maps before being placed in geometrically idealized positions and included as riding atoms with d(C-H) = 0.95 Å and Uiso(H) = 1.2Ueq(C) for arene hydrogen atoms and d(C-H) = 0.99 Å and Uiso(H) = 1.2Ueq(C) for methylene hydrogen atoms. The three unique ammonium hydrogen atoms were located and their coordinates refined. They were assigned a common isotropic displacement parameter. The largest residual electron density peak in the final difference map is 0.72 e^-/Å^3, located 1.32 Å from Cd1.

$((Br)C_{15}H_{15}N)_2CdCl_4$: The compound crystallizes in the orthorhombic system. The pattern of systematic absences in the intensity data was consistent with the space groups *Cmce* and *Aea*2. The non-centrosymmetric group *Aea*2 was found by the solution program SHEXLT and was confirmed by obtaining a reasonable and stable structure solution. The structural model was checked for missed symmetry using ADDSYM (on a hypothetical non-disordered model) which found none.[7–9] The asymmetric unit in *Aea*2 consists of one $C_{15}H_{15}BrN^+$ cation, two chloride anions and (formally) half of one cadmium atom, which is located in a crystallographic two-fold axis of rotation. The $C_{15}H_{15}BrN^+$ is disordered over two orientations, affecting only the inner 'stilbene' atoms. The bromine and –$CH_2NH_3^+$ substituents of the cation are common to both components. The minor component geometry (atom label suffixes "B") was restrained to be similar to that of the major (atom label suffixes "A") using a SHELX SAME instruction (Table S3). The major disorder component occupancy refined to 0.56(1). All non-hydrogen atoms were refined with anisotropic displacement parameters. Some nearly superimposed component atoms of the disordered cation were assigned equal U_{ij} values. Rigid-bond restraints (SHELX RIGU) were used to maintain physically reasonable U_{ij} values for disordered atoms of each component. Further ISOR instructions were used C2, C7 and C13. Hydrogen atoms bonded to carbon were placed in geometrically idealized positions and included as riding atoms with d(C-H) = 0.95 Å and Uiso(H) = 1.2Ueq(C) for arene hydrogen atoms and d(C-H) = 0.99 Å and Uiso(H) = 1.2Ueq(C) for methylene hydrogen atoms. The three unique ammonium hydrogen atoms could be located but were placed in idealized positions with d(N-H) = 0.91 Å and Uiso(H) = 1.2Ueq(N) and allowed to rotate as a rigid group to the orientation of maximum observed electron density. The largest residual electron density peak in the final difference map is 1.36 e^-/Å^3, located 0.79 Å from Cd1. Near convergence, the absolute structure (Flack x) parameter was 0.13(2), consistent with inversion twinning. An inversion matrix was therefore included in the final refinement cycles, with the Flack x parameter representing the minor twin domain volume fraction.

Computational studies

Theoretical calculations were performed on $(C_{15}H_{16}N)_2CdCl_4$ and $((Br)C_{15}H_{15}N)_2CdCl_4$ to provide further insight into their electronic structures. The PDOS calculations used the PBE+D3 functional, while the band structure and effective mass calculations used the HSE06 functional (Tables S6-7). $(C_{15}H_{16}N)_2CdCl_4$ and $((Br)C_{15}H_{15}N)_2CdCl_4$ have direct bandgaps of 3.1 – 3.5 eV (Tables S7). The valence band maxima (VBM) and the conduction band minima (CBM) have dominant contributions by the respective organic molecules (Figure S6). In comparison, the inorganic components in both cases are further away from the Fermi level. These results are in agreement with our previously reported hybrid materials of the same stilbene backbone as the organic component.[4,10]

The bands around the Fermi level are mostly flat (Figures S6a and S6c). The large effective masses (Tables S7) and observed flat bands indicate that both the charge carriers are localized within the lattice. The calculated reduced effective masses ($\mu = (m_e^* * m_h^*)/(m_e^* + m_h^*)$) of these compounds are similar with only a 3% difference between the two. This suggests that the two compounds have similar recombination rates.[11] It should be noted that the incorporation of bromine into the organic molecule in $((Br)C_{15}H_{15}N)_2CdCl_4$ leads to an increased dispersion of the top of the valence band. This confirms the conclusion from the structural analysis that $((Br)C_{15}H_{15}N)_2CdCl_4$ exhibits halogen-halogen interactions. Consequently, the calculated hole effective mass for $((Br)C_{15}H_{15}N)_2CdCl_4$ is much lower than that for $(C_{15}H_{16}N)_2CdCl_4$ (Tables S7). The increased interaction between the organic molecules can have important consequences for light emission efficiency. In metal halides literature, it is known that charge delocalization can lead to reduced emission efficiency.[12–14]

Table S1. Selected single crystal data and structure refinement parameters for **$(C_{15}H_{16}N)_2CdCl_4$**, **$((Br)C_{15}H_{15}N)_2CdCl_4$** and **$C_{15}H_{16}NCl$**.

Formula	**$(C_{15}H_{16}N)_2CdCl_4$**	**$((Br)C_{15}H_{15}N)_2CdCl_4$**	**$C_{15}H_{16}NCl$**
Formula weight(g/mol)	674.77	832.58	245.74
Temperature (K)	100(2)		
Radiation, wavelength (Å)	Mo Kα, 0.71073		
Crystal system	orthorhombic		
Space group	*Pbca*	*Aea*2	*Pca*2$_1$
Z	*4*	*4*	*8*
Unit cell parameters (Å)	a = 7.2632(2)<br>b = 7.4185(2)<br>c = 55.8967(16)	a = 7.4543(7)<br>b = 53.598(5)<br>c = 7.7117(7)	a = 9.0286(17)<br>b = 5.8327(11)<br>c = 48.928(10)
Volume (Å^3)	3011.83(14)	3081.1(5)	2576.6(9)
Density (ρ_{calc}) (g/cm^3)	1.488	1.795	1.267
Absorption coefficient (μ) (mm^{-1})	1.102	3.673	0.273
$\theta_{min} - \theta_{max}$ (°)	5.796 to 52.786	4.56 to 52.87	4.996 to 49.888
Reflections collected	49813	30800	31863
Independent reflections	3070	3162	4509
R^a indices ($I > 2\sigma(I)$)	$R1$ = 0.0521,<br>$wR2$ = 0.1080	$R1$ = 0.0403,<br>$wR2$ = 0.0915	$R1$ = 0.0748,<br>$wR2$ = 0.1968
Goodness-of-fit on F^2	1.211	1.104	1.034
Largest diff. peak and hole (e$^-$/Å^3)	0.72/-0.85	1.36/-1.66	0.56/-0.49

$^{a}R_1 = \sum||F_0| - |F_c||/\sum|F_0|$; $wR_2 = |\Sigma|w(F_0^2 - F_c^2)^2/\sum\left|wF_0^{2\,2}\right|^{1/2}$
where $w = 1/|\sigma^2F_0^2 + (AP)^2 + BP|$, with $P = (F_0^2 + 2F_c^2)/3$ and weight coefficients A and B

Table S2. Fractional atomic coordinates (× 10^4) and equivalent isotropic displacement parameters (U_{eq}) for **$(C_{15}H_{16}N)_2CdCl_4$**.

Atom	*x*	*y*	*z*	U_{eq}, (Å^2)
		**$(C_{15}H_{16}N)_2CdCl_4$**		
Cd1	5000	5000	0	15.34(14)
Cl1	2087.0(15)	7098.3(15)	58.8(2)	17.5(2)
Cl2	5496.4(16)	5177.1(15)	455.0(2)	18.7(3)
N1	5164(6)	9533(5)	423.4(8)	16.5(8)
C1	4361(7)	10530(7)	630.6(8)	21.2(11)
C2	4706(7)	9568(6)	864.5(8)	19.4(10)
C3	3286(8)	8623(7)	972.1(9)	25.8(12)
C4	3564(8)	7745(8)	1192.0(9)	30.9(13)
C5	5259(8)	7850(8)	1306.5(10)	31.2(13)
C6	6651(8)	8820(8)	1198.2(10)	32.6(13)
C7	6398(8)	9663(8)	979.6(9)	29.1(12)
C8	5630(8)	6974(8)	1540.8(10)	34.1(14)
C9	4654(9)	5689(8)	1637.9(10)	33.1(13)
C10	4936(8)	4850(8)	1875.0(9)	28.3(12)
C11	3756(9)	3463(8)	1944.0(10)	33.8(14)
C12	3885(8)	2672(8)	2166.4(10)	35.1(14)
C13	5220(8)	3240(8)	2325.4(9)	29.2(13)
C14	6429(8)	4590(8)	2259.3(9)	30.7(13)
C15	6295(8)	5401(7)	2035.2(9)	28.6(12)

U_{eq} is defined as one-third of the trace of the orthogonalized U_{ij} tensor.

Table S3. Fractional atomic coordinates (× 10^4) and equivalent isotropic displacement parameters (U_{eq}) for **$((Br)C_{15}H_{15}N)_2CdCl_4$**.

Atom	*x*	*y*	*z*	*Ueq*, (Å^2)
		**$((Br)C_{15}H_{15}N)_2CdCl_4$**		
Cd1	5000	0	4934.7(8)	16.1(2)
Cl1	7611(4)	54.8(3)	7385(3)	21.2(4)
Cl2	4548(2)	464.5(4)	5197(3)	20.3(4)
Br1	4906.7(15)	2418.9(2)	-524.7(11)	33.1(3)
N1	4704(9)	393.5(12)	9719(10)	21.3(16)
C1	5407(13)	631.9(18)	10531(13)	28(2)
C2A	5210(40)	847(15)	9280(130)	22(3)
C3A	6690(30)	958(10)	8470(80)	24(4)
C4A	6470(30)	1156(4)	7310(30)	22(3)
C5A	4780(30)	1249(3)	6950(20)	20(4)
C6A	3310(30)	1138(4)	7770(30)	20(3)
C7A	3510(30)	938(7)	8890(60)	22(3)
C8A	4480(20)	1450(3)	5660(20)	22(4)
C9A	5740(20)	1574(3)	4800(20)	27(4)
C10A	5470(30)	1777(3)	3500(20)	23(4)
C11A	6980(30)	1894(4)	2840(30)	27(5)
C12A	6830(30)	2097(6)	1670(40)	23(5)
C13A	5130(30)	2168(7)	1210(50)	25(4)
C14A	3590(30)	2047(8)	1800(60)	31(6)
C15A	3800(30)	1853(4)	3010(30)	27(5)
C2B	5360(50)	851(19)	9310(160)	22(3)
C3B	7010(40)	934(13)	8700(110)	24(4)
C4B	7130(30)	1134(5)	7540(40)	22(3)
C5B	5580(30)	1245(4)	6950(30)	20(4)
C6B	3920(30)	1162(6)	7570(40)	20(3)
C7B	3800(40)	972(9)	8780(80)	22(3)
C8B	5720(30)	1451(4)	5670(30)	26(5)
C9B	4410(30)	1573(3)	4920(30)	32(5)
C10B	4590(40)	1775(4)	3590(30)	23(4)
C11B	6240(30)	1860(5)	3050(40)	26(6)
C12B	6350(40)	2046(7)	1760(60)	26(7)
C13B	4800(40)	2150(9)	1100(70)	25(4)
C14B	3120(40)	2069(9)	1650(70)	24(6)
C15B	3080(40)	1878(6)	2890(40)	27(6)

U_{eq} is defined as one-third of the trace of the orthogonalized U_{ij} tensor.

Table S4. Fractional atomic coordinates (× 10^4) and equivalent isotropic displacement parameters (U_{eq}) for $\mathbf{C_{15}H_{16}NCl}$.

Atom	*x*	*y*	*z*	*Ueq*, (Å^2)
		$\mathbf{C_{15}H_{16}NCl}$		
Cl1	4214.9(19)	9935(3)	4726.7(7)	68.3(9)
Cl2	8109.4(18)	5061(3)	5218.2(7)	65.1(8)
N1	5712(7)	4922(11)	4733(3)	74(3)
C1	6732(11)	4786(14)	4493(3)	87(5)
C2	5928(9)	4101(16)	4238(3)	74(3)
C3	4907(11)	5665(15)	4118(3)	80(3)
C4	4135(14)	4993(14)	3889(3)	75(3)
C5	4299(12)	2836(16)	3776(3)	80(3)
C6	5305(12)	1362(16)	3891(3)	85(4)
C7	6105(10)	1968(15)	4121(3)	92(4)
C8	3428(14)	2051(17)	3539(2)	86(3)
C9	2282(15)	3123(16)	3436(2)	81(3)
C10	1380(16)	2369(16)	3199(2)	85(4)
C11	335(16)	3863(17)	3090(2)	87(4)
C12	-476(19)	3270(20)	2861(2)	105(5)
C13	-280(20)	1178(19)	2740(2)	107(5)
C14	700(20)	-330(20)	2854(3)	112(6)
C15	1550(20)	235(17)	3079(2)	96(5)
N2	6622(7)	62(10)	5204(3)	76(3)
C16	5642(10)	298(17)	5448(3)	83(4)
C17	6480(9)	914(14)	5701(3)	78(3)
C18	6292(11)	3040(15)	5825(3)	84(3)
C19	7046(13)	3656(16)	6054(3)	90(4)
C20	8073(12)	2155(16)	6176(3)	83(3)
C21	8246(14)	-26(15)	6052(4)	85(4)
C22	7471(11)	-548(16)	5826(3)	75(3)
C23	8937(14)	2902(16)	6416(2)	84(3)
C24	10062(13)	1770(18)	6531(2)	78(3)
C25	10905(17)	2397(15)	6777(2)	90(4)
C26	10753(19)	4480(20)	6918(3)	104(5)
C27	11520(20)	4919(18)	7156(3)	115(6)
C28	12510(20)	3370(20)	7257(2)	123(6)
C29	12708(19)	1301(18)	7119(2)	103(4)
C30	11936(17)	876(18)	6885(2)	90(4)

U_{eq} is defined as one-third of the trace of the orthogonalized U_{ij} tensor.

Table S5. A comparison of select bond distances and angles within the anionic units in **$(C_{15}H_{16}N)_2CdCl_4$** and **$((Br)C_{15}H_{15}N)_2CdCl_4$**.

Atom pair	Distance (Å)	Label	Angle (°)
		**$(C_{15}H_{16}N)_2CdCl_4$**	
Cd(1) – Cl(1) × 2	2.6471(11)	Cl(1)– Cd(1) – Cl(1)	180.00(6)
Cd(1) – Cl(1) × 2	2.6533(11)	Cl(1)– Cd(1) – Cl(1)	89.710(20)
Cd(1) – Cl(2) × 2	2.5723(11)	Cl(1)– Cd(1) – Cl(1)	90.290(20)
		Cl(1)– Cd(1) – Cl(1)	90.06(6)
		Cl(2)– Cd(1) – Cl(1)	89.94(6)
		Cl(2)– Cd(1) – Cl(1)	92.34(8)
		Cl(2)– Cd(1) – Cl(1)	87.66(4)
		**$((Br)C_{15}H_{15}N)_2CdCl_4$**	
Cd(1) – Cl(1) × 2	2.728(6)	Cl(1)– Cd(1) – Cl(1)	85.10(12)
Cd(1) – Cl(1) × 2	2.669(6)	Cl(1)– Cd(1) – Cl(1)	176.34(24)
Cd(1) – Cl(1) × 2	2.520(4)	Cl(1)– Cd(1) – Cl(1)	91.283(24)
		Cl(1)– Cd(1) – Cl(1)	92.34(11)
		Cl(2)– Cd(1) – Cl(1)	94.50(12)
		Cl(2)– Cd(1) – Cl(1)	92.27(12)
		Cl(2)– Cd(1) – Cl(1)	86.19(12)
		Cl(2)– Cd(1) – Cl(1)	87.45(6)
		Cl(2)– Cd(1) – Cl(2)	170.80(12)

Table S6. Summary of cell dimensions used in theoretical calculations.

Compound	k-point grid	a (Å)	b (Å)	c (Å)	α (°)	β (°)	γ (°)	Cell Volume (Å^3)
$(C_{15}H_{16}N)_2CdCl_4$ (Unit)	3 × 3 × 1	7.26	7.42	55.90	90	90	90	3011.29
$((Br)C_{15}H_{15}N)_2CdCl_4$ (Unit)	3 × 3 × 1	7.46	53.60	7.71	90	90	90	3082.89
$((Br)C_{15}H_{15}N)_2CdCl_4$ (Primitive)	3 × 3 × 3	27.1	27.08	7.45	90	90	16.38	5463.28

Table S7. Summary of the calculated effective masses and calculated band gaps for **$(C_{15}H_{16}N)_2CdCl_4$** and **$((Br)C_{15}H_{15}N)_2CdCl_4$.**

Systems	Bandgap (eV)		Effective Mass (in units of m_0)		Reduced Effective Mass
Functional	PBE+D3	HSE06	m_e^*	m_h^*	$\mu = \frac{m_e^* m_h^*}{(m_e^*+m_h^*)}$
$(C_{15}H_{16}N)_2CdCl_4$	2.643	3.482	2.957	4.248	1.743
$((Br)C_{15}H_{15}N)_2CdCl_4$	2.302	3.067	5.615	2.643	1.797

Attenuation of high-energy photons

The mass attenuation coefficients for the selected compounds were calculated as the weight-fraction–weighted mean of the elemental mass attenuation coefficients:

$$\mu = \sum_i w_i \, \mu_i$$

where w_i is the weight fraction of element *i* and μ_i is its corresponding mass attenuation coefficient. Energy-dependent elemental mass attenuation coefficients were obtained from the NIST Standard Reference Database 126 (https://www.nist.gov/pml/x-ray-mass-attenuation-coefficients). Because the tabulated NIST data are sampled at non-uniform energy points for different elements, a shape-preserving piecewise cubic interpolation was applied to harmonize the datasets. While this interpolation slightly smooths the step-like features at absorption edges, it does not affect the calculated attenuation length, since the calculations used the tabulated value at 500 keV, which is directly available in the raw data for all the elements. The attenuation length for 500 keV photons was calculated as

$$l_{att}(500keV) = \frac{1}{\rho * \mu_{500keV}}$$

where ρ is the material density. The effective atomic number (Z_{eff}) was estimated using the following empirical expression:

$$Z_{eff} = \sqrt[2.93]{\sum_i \epsilon_i \, Z_i^{2.93}}$$

where ε_i is the electron fraction of element *i* in the compound and Z_i is its atomic number.

Chemical composition	Density [g·cm^3]	Z_{eff} [-]	l_{att}* [cm]
$(C_{15}H_{16}N)_2CdCl_4$	1.49	25	7.3
$((Br)C_{15}H_{15}N)_2CdCl_4$	1.80	27	6.2
$C_{14}H_{12}$ (Stilbene)	0.97	6	11.1
BaF_2	4.89	51	2.1
NaI	3.67	50	2.9
$Bi_4Ge_3O_{12}$ (BGO)	7.11	71	1.0
E_γ = 500 keV			

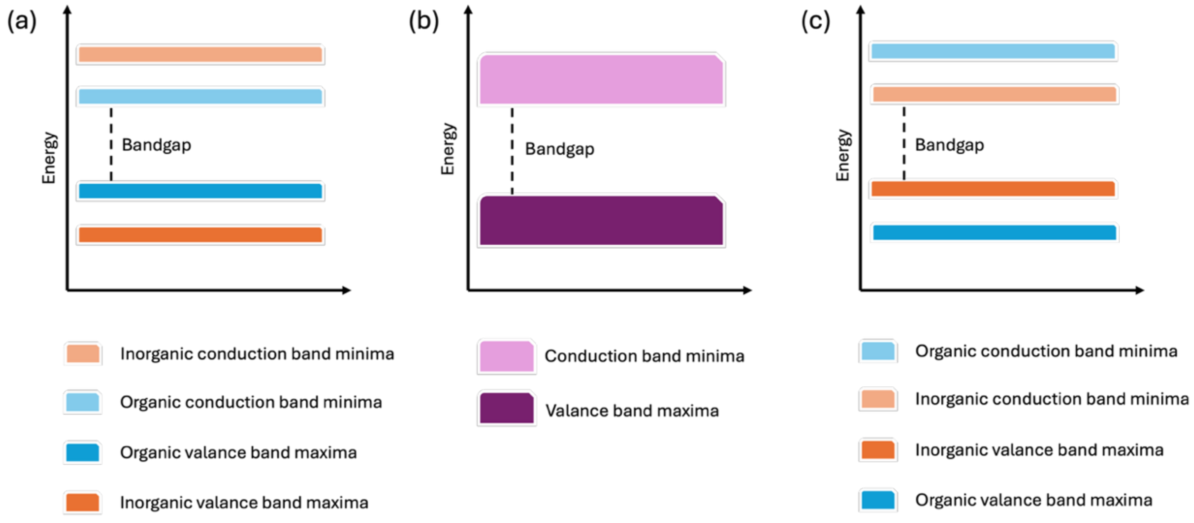

Figure S1. (a) Our materials design in which organic structural units comprise frontier states, in comparison to the frontier states of conventional (b) inorganic semiconductors and (c) hybrid organic-inorganic perovskites.

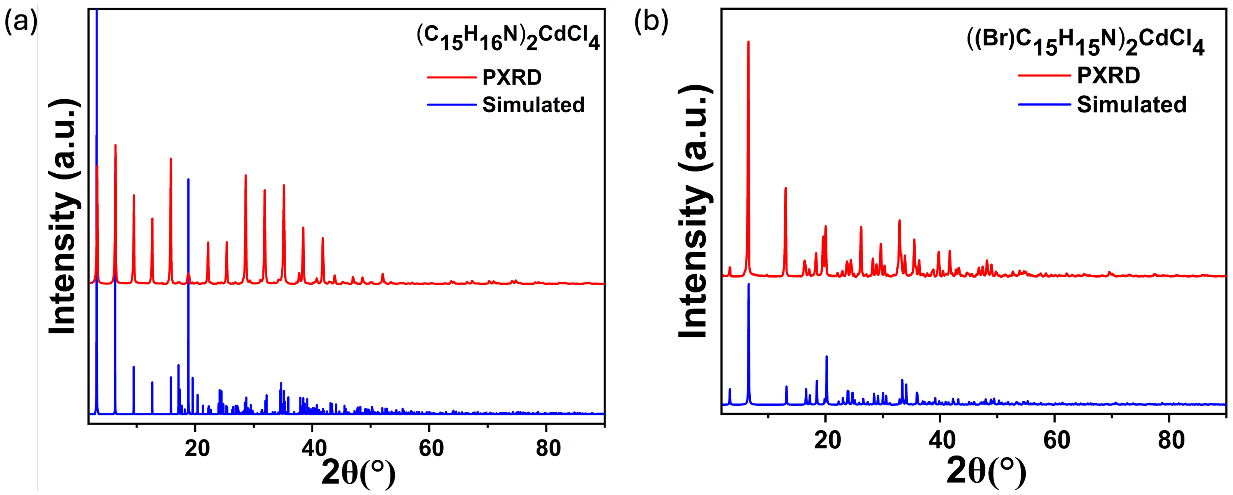

Figure S2. The reported synthesis method yields phase pure samples of (a) $(C_{15}H_{16}N)_2CdCl_4$ and (b) $((Br)C_{15}H_{15}N)_2CdCl_4$ based on PXRD measurements.

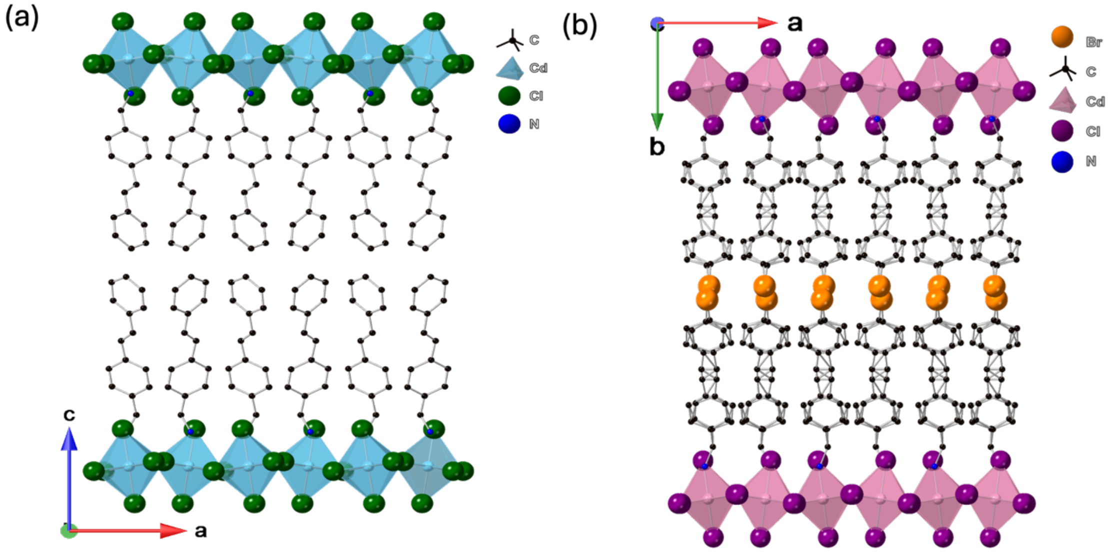

Figure S3. Polyhedral representation of the crystal structure along the *b*-axis in (a) $(C_{15}H_{16}N)_2CdCl_4$ and the *c*-axis in (b) $((Br)C_{15}H_{15}N)_2CdCl_4$.

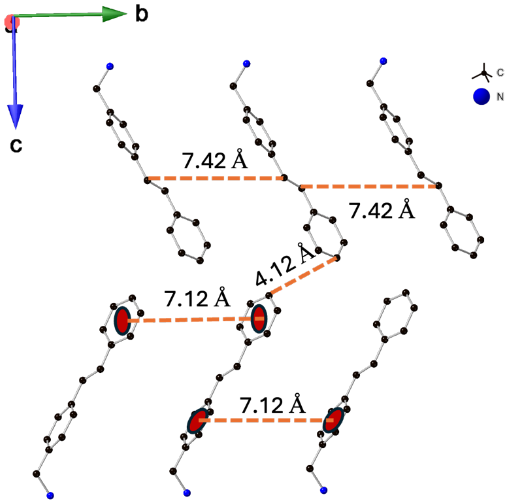

Figure S4. Intermolecular distances in the cationic $(C_{15}H_{16}N)^{+}$ organic layers in $(C_{15}H_{16}N)_2CdCl_4$. The coloring of atoms is identical to that in prior figures.

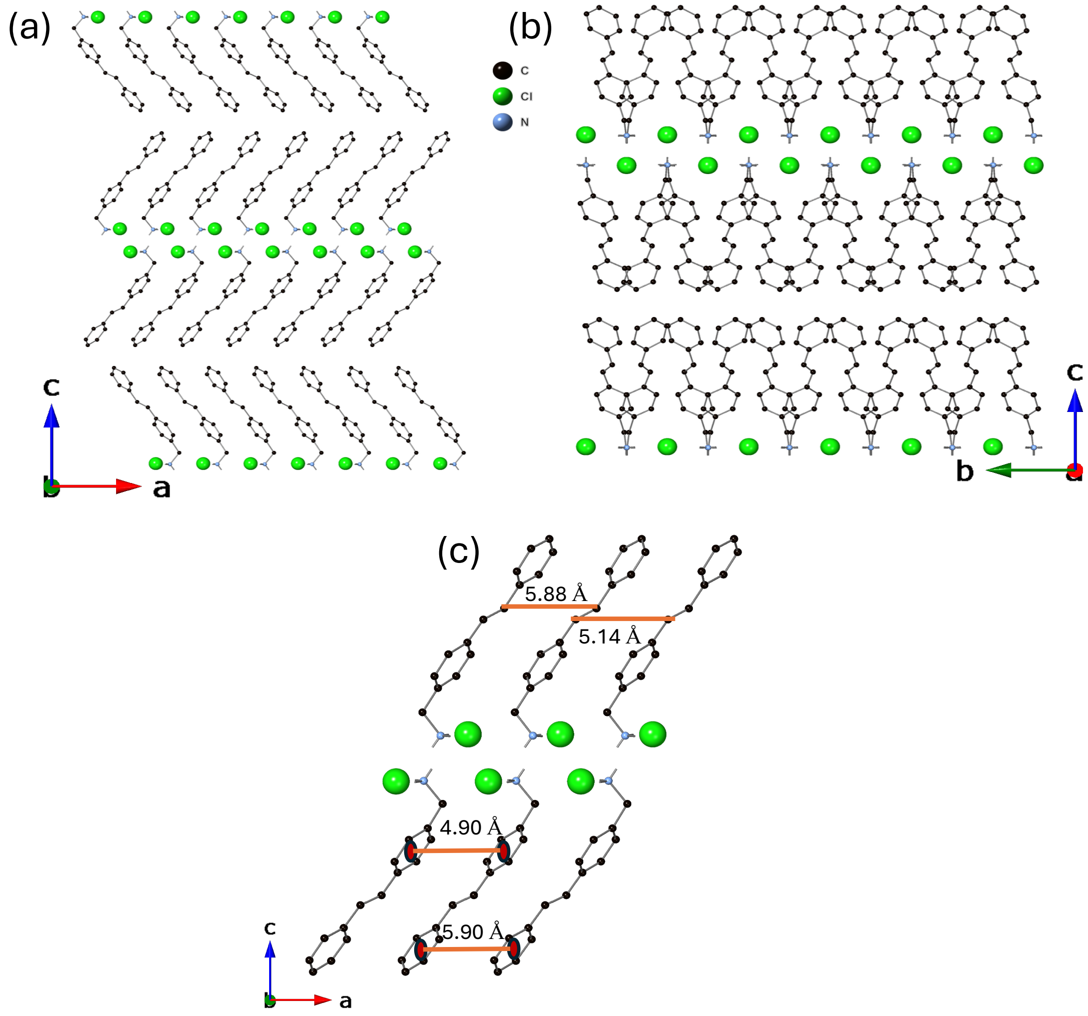

Figure S5. Crystal structure of $C_{15}H_{16}NCl$ viewed along (a) *b*-axis and (b) *a*-axis. Black, green, and blue spheres represent carbon, chlorine, and nitrogen, respectively. For clarity, hydrogen atoms are not depicted. (c) Intermolecular distances in the precursor salt $C_{15}H_{16}NCl$.

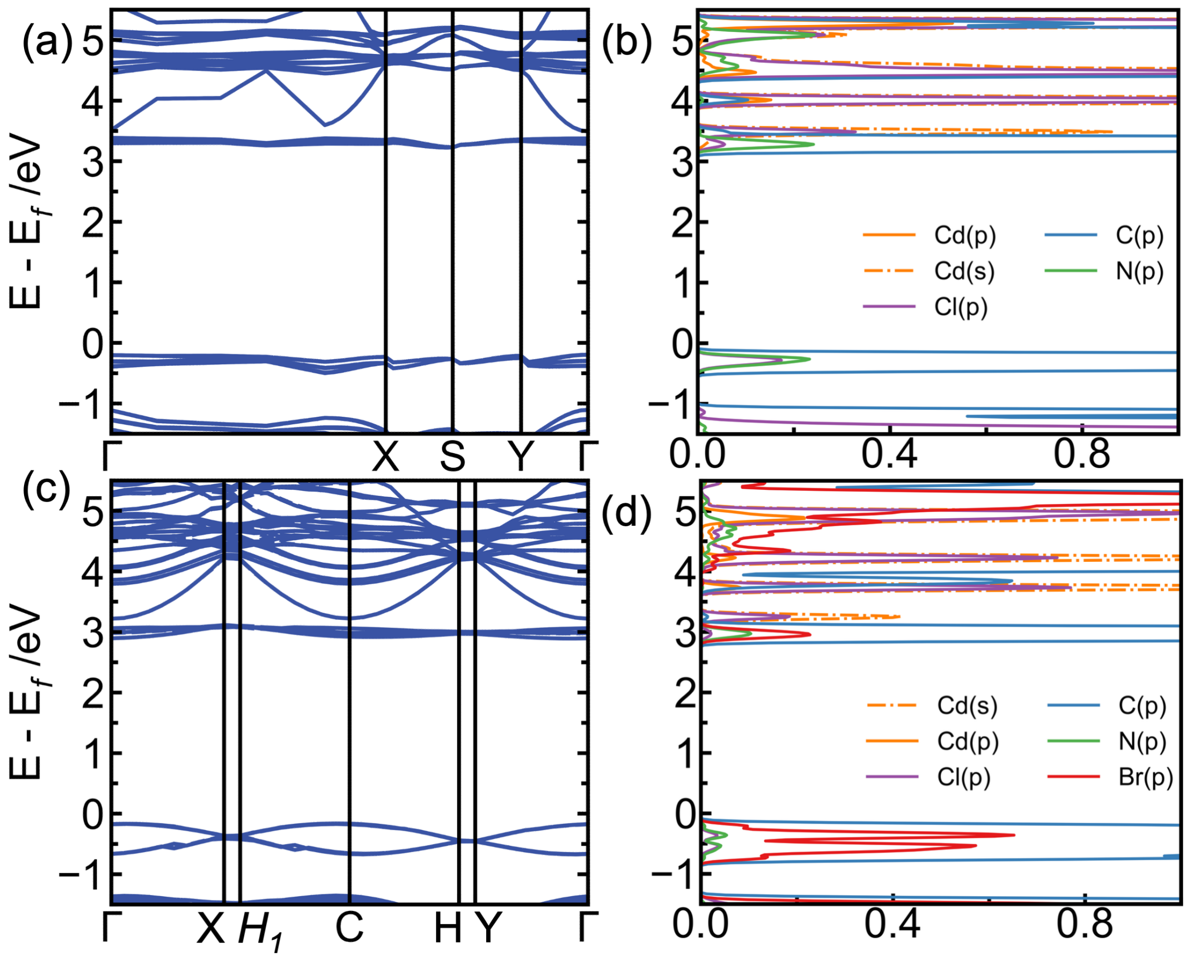

Figure S6. Electronic band structures and projected density of states (PDOS) for (a-b) $(C_{15}H_{16}N)_2CdCl_4$ and (c-d) $((Br)C_{15}H_{15}N)_2CdCl_4$.

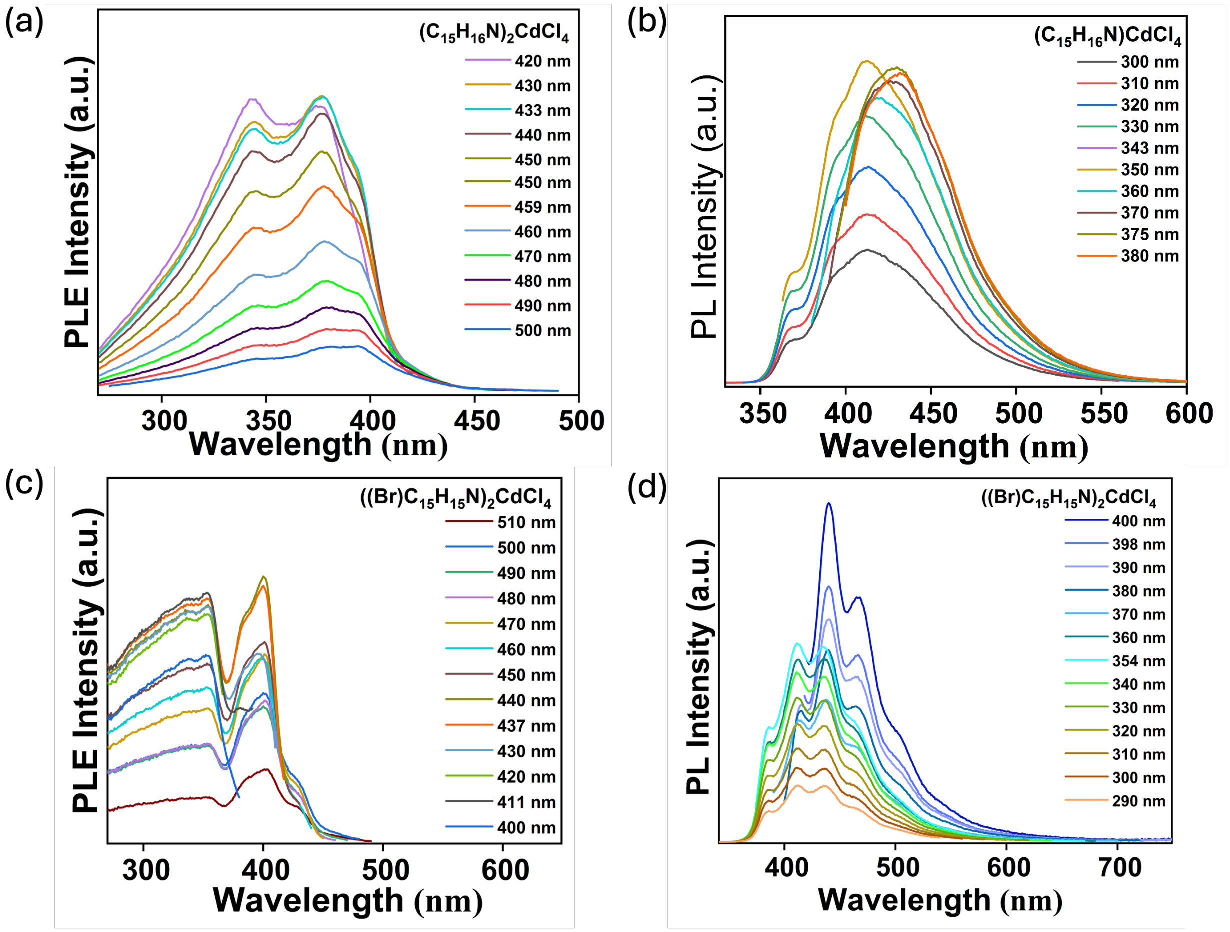

Figure S7. Emission-dependent PLE and excitation-dependent PL spectra of (a-b) $(C_{15}H_{16}N)_2CdCl_4$ and (c-d) $((Br)C_{15}H_{15}N)_2CdCl_4$.

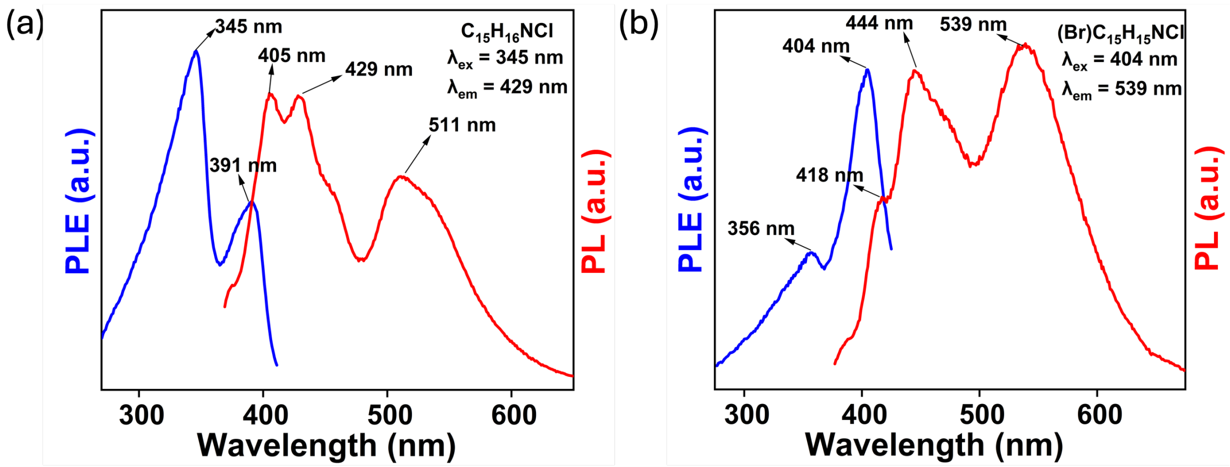

Figure S8. PL and PLE spectra of (a) $C_{15}H_{16}NCl$ and (b) $(Br)C_{15}H_{15}NCl$.

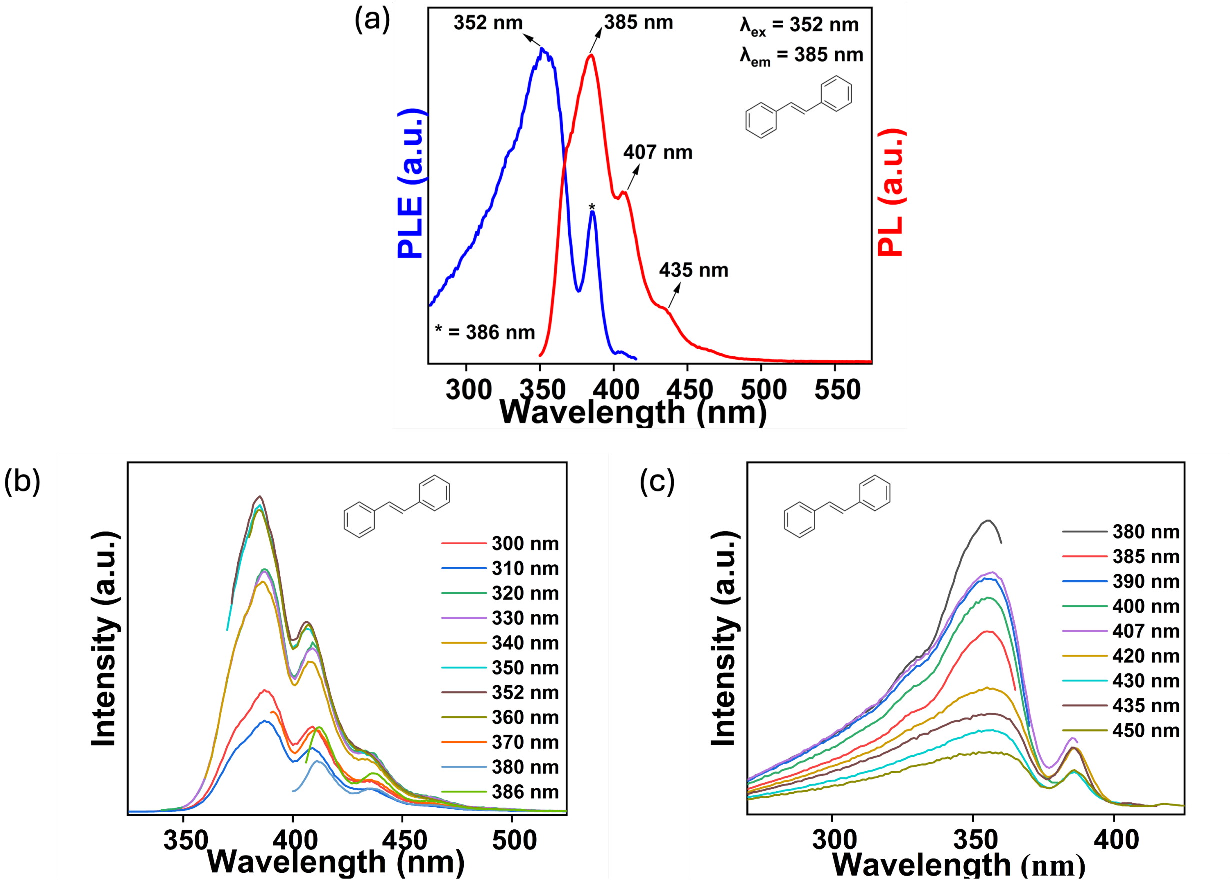

Figure S9. (a) PL and PLE, (b) excitation-dependent PL, and (c) emission-dependent PLE spectra of analytical grade stilbene.

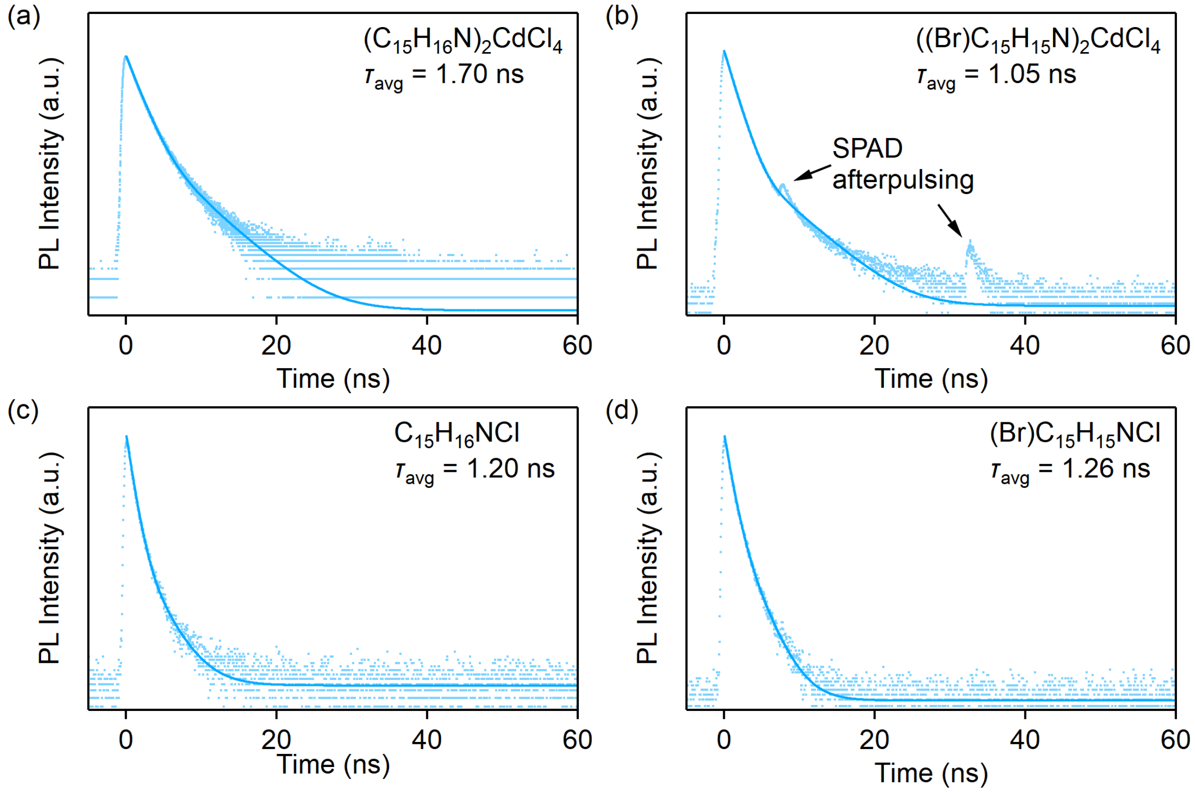

Figure S10. Room temperature time-resolved PL for (a) $(C_{15}H_{16}N)_2CdCl_4$, (b) $((Br)C_{15}H_{15}N)_2CdCl_4$, (c) $C_{15}H_{16}NCl$ and (d) $BrC_{15}H_{15}NCl$. All samples were excited at 405 nm.

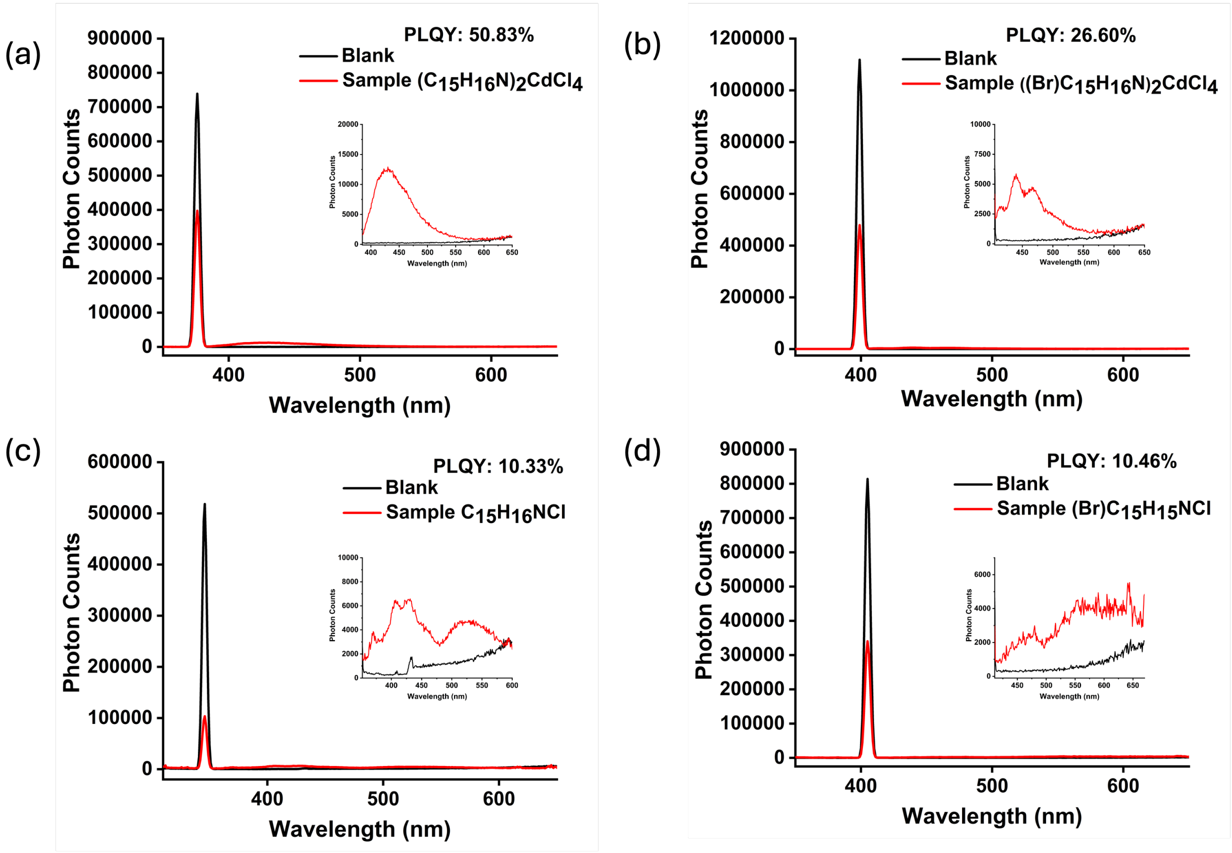

Figure S11. PLQY measurements for (a) $(C_{15}H_{16}N)_2CdCl_4$ (375 nm excitation), (b) $((Br)C_{15}H_{15}N)_2CdCl_4$ (398 nm excitation), (c) $C_{15}H_{16}NCl$ (345 nm excitation) and (d) $(Br)C_{15}H_{15}NCl$ (404 nm excitation). The black lines are for the blank sample holders, while the red lines are the data obtained for the samples.

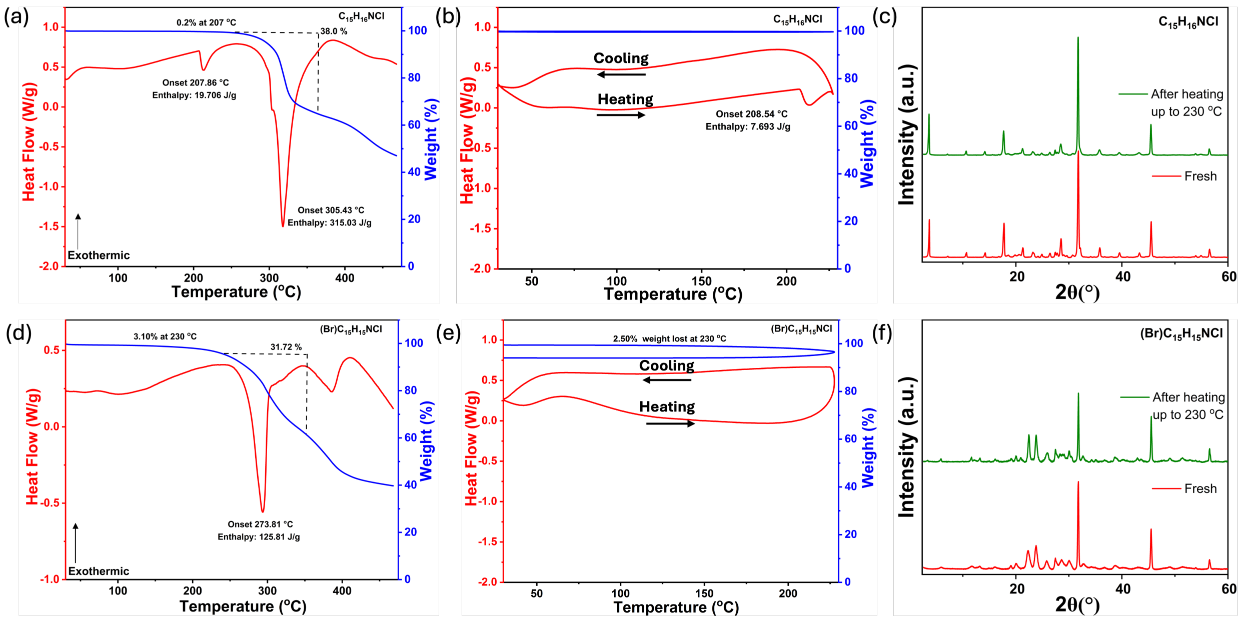

Figure S12. Thermogravimetric analysis (TGA) and differential scanning calorimetry (DSC) measurement results for the precursor organic salts (a) $C_{15}H_{16}NCl$ and (d) $C_{15}H_{16}NCl$ on heating up to 475 °C. TGA/DSC measurements conducted on heating (up to 230 °C) and cooling for (b) $C_{15}H_{16}NCl$ and (e) $BrC_{15}H_{15}NCl$. Powder X-ray diffraction (PXRD) for (c) $C_{15}H_{16}NCl$ and (f) $BrC_{15}H_{15}NCl$ before and after heating up to 230 °C suggest that the observed changes in the RT-230 °C are reversible.

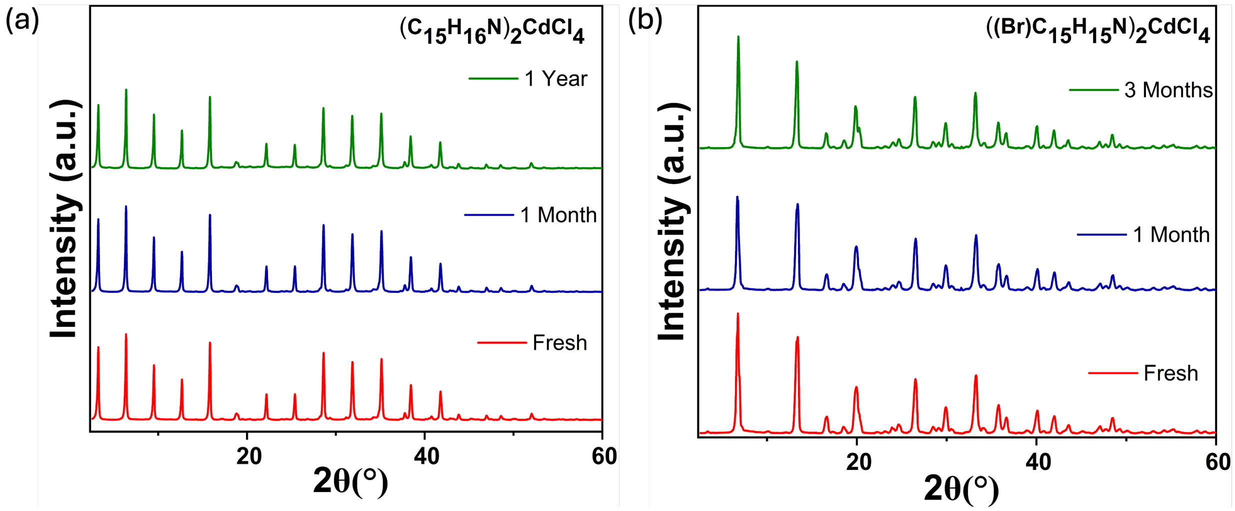

Figure S13. Periodic PXRD measurement results for (a) $(C_{15}H_{16}N)_2CdCl_4$ and (b) $((Br)C_{15}H_{15}N)_2CdCl_4$ samples kept in ambient air over prolonged periods.

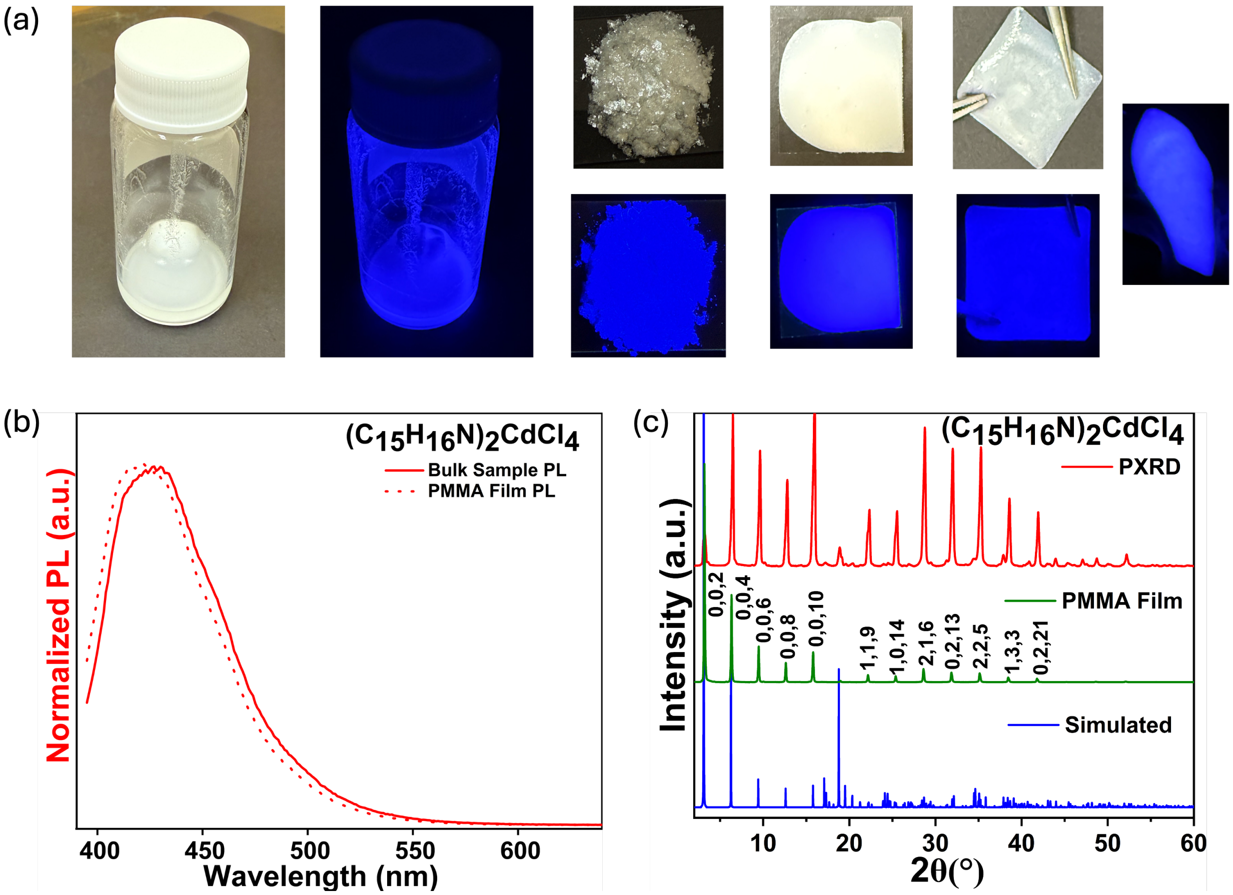

Figure S14. (a) Poly methylmethacrylate (PMMA) composite solution and film of $(C_{15}H_{16}N)_2CdCl_4$ under daylight and 365 nm excitation. (b) Stacked photoluminescence (PL) profiles of the bulk sample and its PMMA composite film from 375 nm excitation. (c) XRD pattern obtained for the resultant PMMA film (green) agrees with that obtained for the bulk sample (red) with simulated data (blue) provided for comparison.

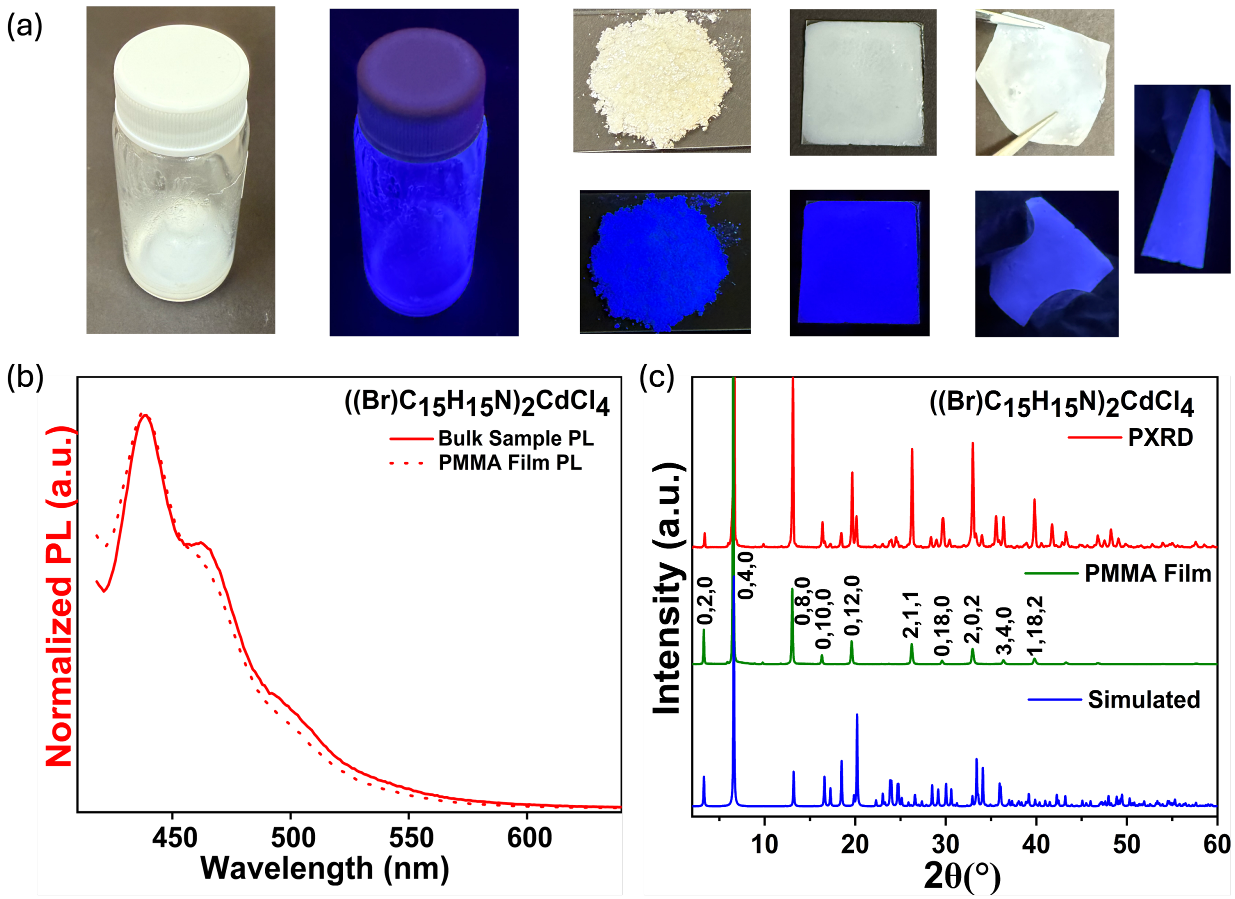

Figure S15. (a) Poly methylmethacrylate (PMMA) composite solution and film of $((Br)C_{15}H_{15}N)_2CdCl_4$ under daylight and 365 nm excitation. (b) Stacked photoluminescence (PL) profiles of the bulk sample and its composite PMMA film from 398 nm excitation. (c) XRD pattern obtained for the resultant PMMA film (green) agrees with that obtained for the bulk sample (red); the simulated pattern (blue) is provided for comparison.

Reference:

(1) Frank, E.; Park, S.; Harrer, E.; Flügel, J. L.; Fischer, M.; Nuernberger, P.; Rehbein, J.; Breder, A. Asymmetric Migratory Tsuji–Wacker Oxidation Enables the Enantioselective Synthesis of Hetero- and Isosteric Diarylmethanes. *J. Am. Chem. Soc.* **2024**, *146* (50), 34383–34393. https://doi.org/10.1021/jacs.4c09405.

(2) D'Imperio, N.; Arkhypchuk, A. I.; Ott, S. *E* , *Z* -Selectivity in the Reductive Cross-Coupling of Two Benzaldehydes to Stilbenes under Substrate Control. *Org. Biomol. Chem.* **2020**, *18* (31), 6171–6179. https://doi.org/10.1039/D0OB01139H.

(3) Chen, Y.; Zhou, L.; Wang, J.; Liu, X.; Lu, H.; Liu, L.; Lv, F.; Wang, S. Photoactive Oligo(*p* -Phenylenevinylene) Functionalized with Phospholipid Units for Control and Visualization of Delivery into Living Cells. *ACS Appl. Mater. Interfaces* **2018**, *10* (33), 27555–27561. https://doi.org/10.1021/acsami.8b07847.

(4) Fattal, H.; Creason, T. D.; Delzer, C. J.; Yangui, A.; Hayward, J. P.; Ross, B. J.; Du, M.-H.; Glatzhofer, D. T.; Saparov, B. Zero-Dimensional Hybrid Organic–Inorganic Indium Bromide with Blue Emission. *Inorg. Chem.* **2021**, *60* (2), 1045–1054. https://doi.org/10.1021/acs.inorgchem.0c03164.

(5) Kon, G. A. R. 56. 4-Styrylbenzylamine and 4-Styrylbenzyldimethylamine. *J. Chem. Soc. Resumed* **1948**, 224. https://doi.org/10.1039/jr9480000224.

(6) Nikovskiy, I. A.; Isakovskaya, K. L.; Nelyubina, Y. V. New Low-Dimensional Hybrid Perovskitoids Based on Lead Bromide with Organic Cations from Charge-Transfer Complexes. *Crystals* **2021**, *11* (11), 1424. https://doi.org/10.3390/cryst11111424.

(7) Palatinus, L.; Van Der Lee, A. Symmetry Determination Following Structure Solution in *P* 1. *J. Appl. Crystallogr.* **2008**, *41* (6), 975–984. https://doi.org/10.1107/S0021889808028185.

(8) Spek, A. L. *LEPAGE* – an MS-DOS Program for the Determination of the Metrical Symmetry of a Translation Lattice. *J. Appl. Crystallogr.* **1988**, *21* (5), 578–579. https://doi.org/10.1107/S002188988800490X.

(9) Spek, A. L. Structure Validation in Chemical Crystallography. *Acta Crystallogr. D Biol. Crystallogr.* **2009**, *65* (2), 148–155. https://doi.org/10.1107/S090744490804362X.

(10) Creason, T. D.; Fattal, H.; Gilley, I. W.; Evans, B. N.; Jiang, J.; Pachter, R.; Glatzhofer, D. T.; Saparov, B. Stabilized Photoemission from Organic Molecules in Zero-Dimensional Hybrid Zn and Cd Halides. *Inorg. Chem. Front.* **2022**, *9* (23), 6202–6210. https://doi.org/10.1039/d2qi01293f.

(11) Popy, D. A.; Singh, Y.; Tratsiak, Y.; Cardoza, A. M.; Lane, J. M.; Stand, L.; Zhuravleva, M.; Rai, N.; Saparov, B. Stimuli-responsive Photoluminescent Copper(I) Halides for Scintillation, Anticounterfeiting, and Light-emitting Diode Applications. *Aggregate* **2024**, e602. https://doi.org/10.1002/agt2.602.

(12) Li, S.; Luo, J.; Liu, J.; Tang, J. Self-Trapped Excitons in All-Inorganic Halide Perovskites: Fundamentals, Status, and Potential Applications. *J. Phys. Chem. Lett.* **2019**, *10* (8), 1999–2007. https://doi.org/10.1021/acs.jpclett.8b03604.

(13) Heckelmann, I.; Lu, Z.; Prentice, J. C. A.; Auras, F.; Ronson, T. K.; Friend, R. H.; Nitschke, J. R.; Feldmann, S. Supramolecular Self-Assembly as a Tool To Preserve the Electronic Purity of Perylene Diimide Chromophores**. *Angew. Chem. Int. Ed.* **2023**, *62* (12), e202216729. https://doi.org/10.1002/anie.202216729.

(14) Ray, A.; De Trizio, L.; Zito, J.; Infante, I.; Manna, L.; Abdelhady, A. L. Light Emission from Low-Dimensional Pb-Free Perovskite-Related Metal Halide Nanocrystals. *Adv. Opt. Mater.* **2023**, *11* (4), 2202005. https://doi.org/10.1002/adom.202202005.
